# A general and facile one-pot process of isothiocyanates from amines under aqueous conditions

**DOI:** 10.3762/bjoc.8.6

**Published:** 2012-01-10

**Authors:** Nan Sun, Bin Li, Jianping Shao, Weimin Mo, Baoxiang Hu, Zhenlu Shen, Xinquan Hu

**Affiliations:** 1 College of Chemical Engineering and Materials Science, Zhejiang University of Technology, Hangzhou 310014, China; 2 State Key Laboratory for Oxo Synthesis and Selective Oxidation, Lanzhou Institute of Chemical Physics, Chinese Academy of Sciences, Lanzhou 730000, China

**Keywords:** aqueous conditions, cyanuric acid, isothiocyanates, one-pot process, organic synthesis, primary amines

## Abstract

A general and facile one-pot protocol for the preparation of a broad range of alkyl and aryl isothiocyanates has been developed from their corresponding primary amines under aqueous conditions. This synthetic process involves an in situ generation of a dithiocarbamate salt from the amine substrate by reacting with CS_2_ followed by elimination to form the isothiocyanate product with cyanuric acid as the desulfurylation reagent. The choice of solvent is of decisive importance for the successful formation of the dithiocarbamate salt particularly for highly electron-deficient substrates. This novel and economical method is suitable for scale-up activities.

## Introduction

Isothiocyanates are a class of heteroallenic compounds which are abundant in many cruciferous vegetables. Recently, it has been found that naturally occurring isothiocyanates play a significant role in the cancer chemopreventive activity of these plant species [1–2], and thus, many of such analogues containing the isothiocyanate motif have been synthesized for potential medical applications [3–6]. Morevoer, isothiocyanates are pivotal intermediates in organic synthesis, especially in the synthesis of various heterocyclic compounds [7–8] and unsymmetric thioureas [9–12]. Although many synthetic methods for the preparation of isothiocyanates have been reported to date [13–33], most methods suffer from the employment of highly toxic reagents such as thiophosgene and its analogs, the availability of non-commercialized reagents, or from a narrow substrate scope. Recently, a two-step approach, which was named reagent-promoted desulfurylation of dithiocarbamates strategy, has attracted much attention. In the context of this method, an amine is typically converted into a corresponding dithiocarbamate by reacting with CS_2_ in the presence of a base; and a subsequent desulfurylation affords the desired isothiocyanate with proper desulfurylation reagent. Although many desulfurylating reagents for this strategy were developed [34–59], most of them were efficient only for alkyl and electron-rich aryl isothiocyanates. Few efficient methods were reported for those substrates with highly electron-withdrawing groups (such as, CF_3_, CN, CH_3_CO or NO_2_). Therefore, the development of a more efficient method for the synthesis of isothiocyanates – particularly highly electron-deficient aryl isothiocyanates – is still a challenge in organic chemistry.

## Results and Discussion

Previously, Furumoto reported the application of cyanuric chloride (2,4,6-trichloro-1,3,5-triazine, TCT) as a desulfurylation reagent in the synthesis of carbodiimides from thioureas under mild conditions [60]. In that reaction, the *S*-nucleophiles first reacted with TCT and then decomposed to release the product carbodiimides and by-product 2,4,6-trimercapto-1,3,5-triazine (TMT) [61]. Considering that TCT is an efficient desulfurylation reagent of thioureas to synthesize carbodiimides and that it is affordable in large scale, we speculated that it could also be effective and practical in the desulfurylation of dithiocarbamates to form isothiocyanates.

**Scheme 1:**
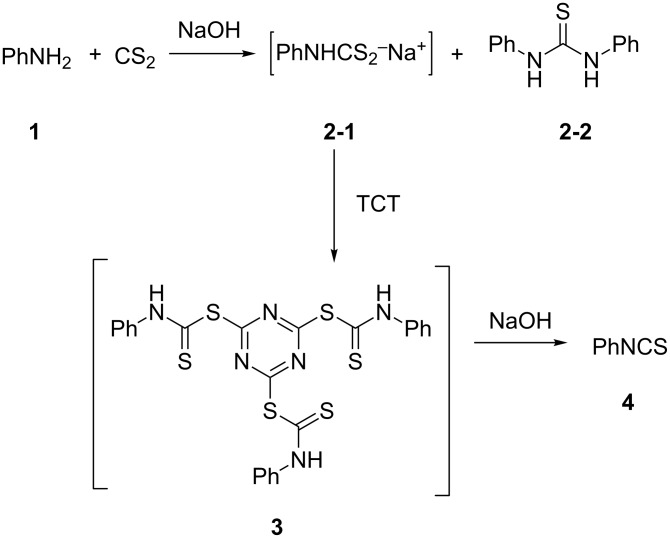
The proposed process for the formation of *N*-phenyl isothiocyanate from aniline.

Our initial study started with aniline (**1**, Scheme 1) to prepare phenyl isothiocyanate (**4**, Scheme 1) via *N*-phenyl dithiocarbamate (**2-1**, Scheme 1) followed by desulfurylation with TCT. Because inorganic bases were more efficient during the conversion of arylamines to *N*-aryl dithiocarbamates in aqueous systems [54–59], we chose inorganic bases to screen the optimal reaction conditions for the one-pot synthesis of **4**. Following the literature procedure, *N*-phenyl dithiocarbamate was generated by mixing aniline and 2.0 equiv of CS_2_ (based on aniline) in water with 1.0 equiv of NaOH at room temperature. After the complete disappearance of the aniline, the mixture was cooled to 0 °C and a solution of TCT (0.5 equiv) in CH_2_Cl_2_ was added. Then the biphasic mixture was stirred for 0.5 h and basified by addition of 6 N NaOH to form a clear solution. A usual work-up afforded the phenyl isothiocyanate in 61% isolated yield along with a small amount of *N*,*N*’-diphenylthiourea (Table 1, entry 1). We proposed that the overall reaction proceeded as described in Scheme 1.

**Table 1:** Reaction of aniline with CS_2_ in aqueous base solutions^a^.

entry	base	aniline/CS_2_/base (equiv)	time (h)	conv^b^ (%)	select^b^ (%) **2-1**:**2-2**	TCT^c^ (equiv)	yield^d^ (%)

1	NaOH	1:2:1	3	94	63:35	0.5	61
2	KOH	1:2:1	3	93	72:26	0.5	63
3	NH_4_OH^e^	1:2:1	5	70	95:2	–	–
4	Na_2_CO_3_	1:2:1	5	86	90:6	–	–
5	NaHCO_3_	1:2:1	5	57	89:2	–	–
6	K_2_CO_3_	1:2:1	5	86	97:1	–	–
7	K_2_CO_3_	1:2:2	3	>99	99:0	0.5	97
8	K_2_CO_3_	1:1.2:2	3	>99	99:0	0.5 0.35	98 89

^a^Reaction conditions: 20 mmol of aniline was used in the reaction of the formation of *N*-phenyl dithiocarbamate at room temperature. The desulfurylation of in situ generated *N*-phenyl dithiocarbamate was carried out by dropwise addition of a solution of TCT in CH_2_Cl_2_ at 0 °C and then, the mixture was stirred for another 0.5 h followed by basification to pH >11 with 6 N NaOH. ^b^Determined by HPLC. ^c^The amount of TCT was based on aniline. ^d^Isolated yield. ^e^28% aqueous NH_4_OH was used.

The success observed in our preliminary study encouraged us to further optimize the conditions for better yields. Our immediate effort was to enhance the selectivity of *N*-phenyl dithiocarbamate over 1,3-diphenylthiourea (**2-2**) at the first stage. Although the formation of *N*-phenyl dithiocarbamate by reaction of aniline and CS_2_ in the presence of inorganic base under aqueous conditions has already been reported, the conversion of aniline and the selection of *N*-phenyl dithiocarbamate have not been disclosed before. Thus, with the help of HPLC analysis, the selectivity of *N*-phenyl dithiocarbamate (**2-1** in Scheme 1) was first studied by testing the base effect. The results of the selectivity of **2-1** with various bases are summarized in Table 1. As can be seen from Table 1, the conversion and the selectivity were influenced by the strength of the bases. When strong bases – such as NaOH and KOH – were employed, a significant amount of 1,3-diphenylthiourea (**2-2**) was formed; this resulted in the low selectivity for *N*-phenyl dithiocarbamate (**2-1**) (Table 1, entry 1 and 2) despite a high conversion observed for the anilines. When weaker bases – such as NH_4_OH, Na_2_CO_3_ and NaHCO_3_ – were used, the selectivity of *N*-phenyl dithiocarbamate was obviously improved but the conversion of aniline decreased even at elongated reaction time (Table 1, entries 3–5). A high selectivity for **2-1** (97:1) along with high conversion (86%) was achieved (Table 1, entry 6) when K_2_CO_3_ was used. When the reaction was carried out with 2 equiv of K_2_CO_3_, a complete conversion was observed. More importantly, the selectivity of **2-1** over **2-2** was almost exclusive (Table 1, entry 7). Further studies indicated that only a slight excess of CS_2_ is needed for a high conversion (Table 1, entry 8).

With an efficient method for the formation of **2-1** in hand, we then continued our research on the synthesis of phenyl isothiocyanate by the desulfurylation of **2-1** with TCT. We found out that the reaction between *N*-phenyl dithiocarbamate and TCT proceeded rather fast, even at a lower temperature (0 °C), and 0.5 equiv of TCT was proved to be necessary for a complete conversion of *N*-phenyl dithiocarbamate. It turned out that the decomposition of the adduct formed between *N*-phenyl dithiocarbamate and TCT to release phenyl isothiocyanate was incomplete under neutral to weak basic conditions. However, when the reaction mixture was basified to pH >11 with 6 N NaOH, a complete decomposition of the adduct was observed. Moreover, under this conditions, the by-product TMT was easily soluble in water to form a clear solution and thus, convenient to the layers separation during the workup. Under optimal conditions, an overall yield of phenyl isothiocyanate from aniline was obtained up to 98% (Table 1, entry 8). It should be noted that choosing K_2_CO_3_ as the reaction base was crucial. It was found out that only the potassium salt of *N*-phenyl dithiocarbamate could form a clear aqueous solution, while the corresponding sodium salt was much less soluble and the ammonium salt was almost insoluble and difficult to stir. Although the isolation of intermediate **3** was not successful (Scheme 1), the fact that the formation of the side product TMT was observed strongly indicated the presence of this intermediacy.

To further study the substrate scope of this newly developed method, various amines were subjected to the treatment with CS_2_ in aqueous K_2_CO_3_ solution followed by desulfurylation with TCT under optimal conditions (1.2 equiv of CS_2_, 2.0 equiv of K_2_CO_3_ at room temperature, and 0.5 equiv of TCT in CH_2_Cl_2_ at 0 °C). The results are summarized in Table 2. As can be seen from Table 2, all tested alkylamines and electron-rich arylamines afforded their corresponding isothiocyanates in excellent yields (Table 2, entries 1–13). Compared to arylamines, alkylamines can generally be converted into their corresponding dithiocarbamates in a relative short time. This rate difference could be originated from the different basicities and their differences in water solubility. Once the dithiocarbamate was generated, the desulfurylation step using TCT proceeded smoothly for all amines. The branched alkylamines, cyclic alkylamines and benzylamines showed similar activity as linear alkylamines (Table 2, entries 1–9). The relative low yields of isopropyl isothiocyanate and *tert*-butyl isothiocyanate were ascribed to their volatility loss associated with isolation (Table 2, entries 1 and 3). The substituent pattern on the phenyl ring of arylamines exhibited no different activity. This also illustrated the tolerance of some steric bulk in the synthesis of isothiocyanate (Table 2, entries 11–13). Under similar reaction conditions, we then tested some electron-deficient arylamines. The difficulty in the formation of their potassium salts of dithiocarbamate was not surprising. The reaction required a slightly higher temperature and a larger excess of CS_2_ to complete the conversion of substrate amines. For example, 3 equiv of CS_2_ was needed for the conversion of 4-fluoroaniline at 40 °C in 12 h during the first step. When *N*-(4-fluorophenyl)dithiocarbamate was formed, 4-fluorophenyl isothiocyanate could be smoothly obtained in reasonable yield after treatment with TCT. However, under the same reaction conditions as 4-fluoroaniline, the reaction rate of 4-chloroaniline was much slower and a longer reaction time was needed. Increasing the amount of CS_2_ did not improve the conversion. Increasing the reaction temperature to 60 °C did improve the conversion of 4-chloroaniline but generated a lot of impurities. With 3 equiv of CS_2_ at 40 °C for 20 h, about 80% conversion of 4-chloroaniline with 92% selectivity of dithiocarbamate was observed. However, only 70% yield of 4-chlorophenyl isothiocyanate was obtained (Table 2, entry 15). The more electron-deficient arylamines, such as 4-trifluoromethyl- and 4-cyanoaniline, failed to convert into their corresponding isothiocyanates when the same procedure as described for 4-chloroaniline was applied.

**Table 2:** Preparation of isothiocyanates^a^.

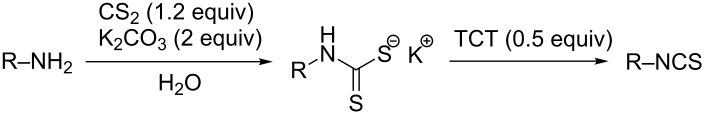				

entry	substrate	product	time^b^ (h)	yield^c^ (%)

1	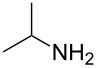	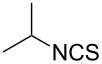	1	85
2	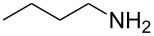	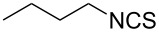	1	94
3	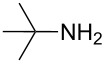	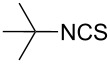	1	80
4	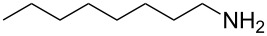	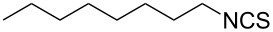	1	95
5	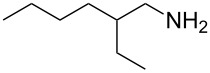	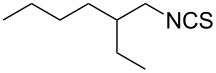	1	98
6	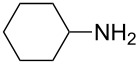	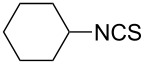	1	95
7	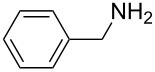	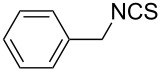	1	99
8	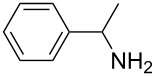	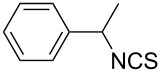	1	99
9	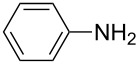	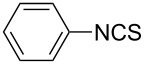	3	98 (94)
10	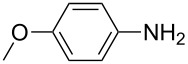	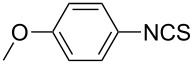	3	86
11	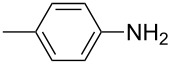	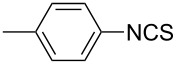	3	93
12	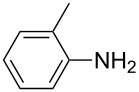	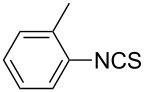	3	95
13	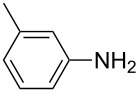	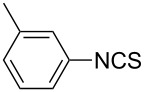	3	98
14	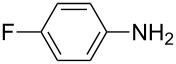	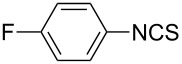	12	81^d^
15	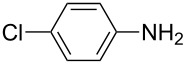	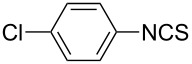	20	70^d^

^a^Reaction conditions: 20 mmol of amine substrate, 24 mmol of CS_2_, 40 mmol of K_2_CO_3_, room temperature. HPLC monitored the conversion. After the amine was totally consumed, the mixture was cooled to 0 °C, and 10 mmol of TCT in CH_2_Cl_2_ was added dropwise. The mixture was stirred for another 0.5 h and basified to pH >11 with 6 N NaOH. ^b^The reaction time for the first step. ^c^Isolated yield. Data in the parenthesis is the isolated yield in 1-mol scale. ^d^3.0 equiv of CS_2_ was used and the reaction temperature was 40 °C.

Moreover, to evaluate the procedure for the potential scaling-up capability, we used aniline as test substrate to scale up the synthesis in 1.0 mol scale. It became clear that the desired phenyl isothiocyanate could be conveniently isolated via vacuum distillation (Table 2, entry 9). This experiment demonstrated its potential for a larger-scale application.

The inefficiency observed for the highly electron-deficient arylamines drove us to further optimize the process. We speculated that the inefficiency for those strongly electron-deficient arylamines could be ascribed to the difficulty in the generation of dithiocarbamates between amines and CS_2_ in aqueous K_2_CO_3_ solution. This prompted us to seek new reaction conditions. It was reported that C_2_H_5_OH was a beneficial solvent in the conversion of electron-deficient arylamines to their corresponding dithiocarbamates with concentrated NH_4_OH [54]. The effect of C_2_H_5_OH in reaction media might facilitate the solubility of arylamines in water and thus, accelerate the reaction. Unfortunately, we found that CS_2_ would react with C_2_H_5_OH to form insoluble solid when it was charged into the reaction mixture. This solid was separated and confirmed to be potassium ethylxanthate formed between CS_2_ and C_2_H_5_OH in aqueous basic solution [62]. On the other hand, much better results were observed when acetonitrile, DMF or DMAc was employed. For example, with 4-chloroaniline as the substrate (Table 3), the results showed that the reaction rate of 4-chloroaniline with CS_2_ in the aqueous K_2_CO_3_ solution was significantly improved with acetonitrile, DMF or DMAc, and best conversion was obtained with DMF as co-solvent (Table 3). The optimized result showed that a solvent mixture of DMF/water (1:4) is suitable for electron-deficient arylamines (Table 3, entry 5).

**Table 3:** Preparation of 4-chlorophenyl isothiocyanate in different solvents^a^.

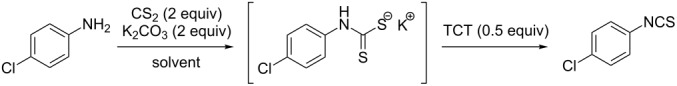				

entry	solvent (v/v)^b^	time (h)^c^	conversion (%)^d^	yield (%)^e^

1	H_2_O	20	56	50
2	CH_3_CN:H_2_O (1:2)	20	80	64
3	DMAc:H_2_O (1:2)	6	91	86
4	DMF:H_2_O (1:2)	6	98	91
5	DMF:H_2_O (1:4)	6	98	92
6	DMF:H_2_O (1:6)	10	90	80

^a^Reaction conditions: 20 mmol of 4-chloroaniline was used in the reaction of the formation of *N*-(4-chlorophenyl)dithiocarbamate at 40 °C. The desulfurylation of in situ generated *N*-(4-chlorophenyl)dithiocarbamate was carried out by dropwise addition of a solution of TCT in CH_2_Cl_2_ at 0 °C and then the mixture was stirred another 0.5 h followed by basification to pH >11 with 6 N NaOH. DMAc = *N,N*-dimethyl acetamide. ^b^20 mL of H_2_O used. ^c^The reaction time for the conversion of 4-chloroaniline to potassium *N*-(4-chlorophenyl)dithiocarbamate. ^d^Determined by HPLC. ^e^Isolated yield.

Under optimal conditions, various electron-deficient arylamines were converted into the desired isothiocyanates in high yield and the results were listed in Table 4. With the help of DMF, more CS_2_ and longer reaction time, those strong electron-deficient arylamines, for examples, CF_3_, CN, CH_3_CO-substituted arylamines, could be also converted into their dithiocarbamates, which could smoothly afford high yield of their corresponding isothiocyanates by further treatment with TCT (Table 4, entries 5–7). To our surprise, in the case of 4-nitroaniline as the substrate, only 13% of 4-nitrophenyl isothiocyanate was obtained along with up to 50% of 1,4-diisothiocyanatobenzene (Table 4, entry 8). This probably resulted from the partial reduction of 4-nitroaniline to 1,4-diaminobenzene by CS_2_ in aqueous K_2_CO_3_ solution.

**Table 4:** Preparation of electron-deficient aryl isothiocyanates^a^.

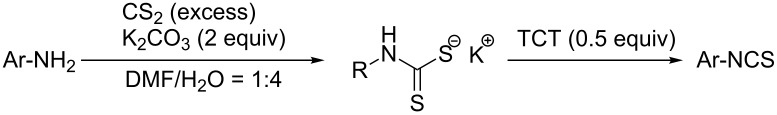					

Entry	Substrate	Product	CS_2_ (equiv)	Time ^b^ (h)	Yield ^c^ (%)

1	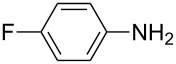	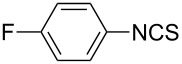	1.2	3	94
2	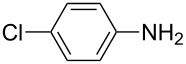	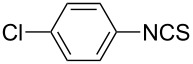	2	6	92
3	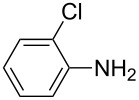	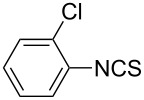	2	6	90
4	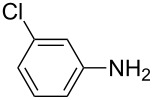	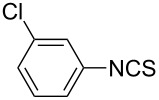	2	6	89
5	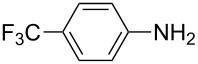	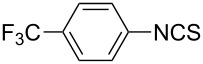	5	7	89
6	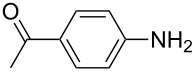	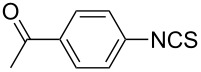	5	10	86
7	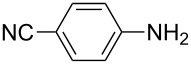	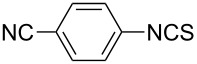	5	10	91
8	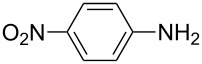	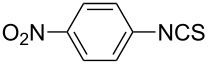	10	72	13
		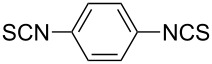			50

^a^Reaction conditions: The substrate (20 mmol) was treated with excess of CS_2_ and 2 equiv of K_2_CO_3_ in the mixture of 5 mL of DMF and 20 mL of H_2_O at 40 °C. After the conversion of the substrate was complete, the mixture was cooled to 0 °C and 10 mmol of TCT in CH_2_Cl_2_ was added dropwise. The mixture was stirred for another 0.5 h and basified to pH >11 with 6 N NaOH. ^b^The reaction time for the conversion of amine to the corresponding dithiocarbamate. ^c^Isolated yield.

## Conclusion

In summary, we have successfully developed a facile and general method to synthesize isothiocyanates from amines. In the context of this method, the amines reacted with CS_2_ in aqueous K_2_CO_3_ solutions to afford dithiocarbamate intermediates, which were further desulfurylized with TCT at 0 °C to provide the corresponding isothiocyanates. The newly developed method could convert a wide range of primary alkyl and arylamines into their corresponding isothiocyanates in excellent yields and provides promise for further scale-up activities. Morevover, this method is advantageous over many other methods for the synthesis of highly electron-deficient aromatic isothiocyanates.

## Experimental

### General

All solvents and reagents were purchased from commercial sources and used without further purification. Melting points were measured without correction. Proton NMR spectra were recorded on a 500 MHz spectrometer in CDCl_3_ with tetramethylsilane (TMS) as internal standard. IR spectra were performed on a FTIR instrument. GC–MS analyses were performed in EI mode. HPLC analyses were performed using a Atlantis ODS column (150 × 4.6 mm (i.d.), 3.5 μm) with a UV detector (280 nm) at room temperature and mobile phase (60:40 CH_3_CN/5 mmol/L K_2_HPO_4_ water solution) with a flow rate of 0.8 mL/min. GC analyses were performed with a FID detector using a HP-5 capillary column (30 m × 0.25 mm (i.d.), 0.25 μm film thickness). The flow rate of carrier gas (N_2_) was 1.0 mL/min with a split ratio of 50:1. The column temperature was increased from an initial temperature of 100 to 280 °C at 10 °C/min and maintained at this temperature for 10 min. The FID detector temperature was kept at 280 °C. All operations were carried out under atmospheric conditions and all synthesized isothiocyanates were stable under the operational conditions.

#### Typical procedure for the preparation of alkyl and aryl isothiocyanates

To a mixture of amine (20 mmol) and K_2_CO_3_ (5.52 g, 40 mmol) in 20 mL of water 1.82 g of CS_2_ (24 mmol) was added dropwise in a period of 20–30 min at room temperature. After the addition was complete, the mixture was stirred for several hours until complete conversion was determined by HPLC (arylamine) or GC (alkylamine). Then, the reaction mixture was cooled to 0 °C and a solution of 1.85 g of TCT (10 mmol) in 15 mL of CH_2_Cl_2_ was added dropwise. After the addition was complete, the mixture was stirred for another 0.5 h to finish the reaction. The reaction mixture was then basified to pH >11 with 6 N NaOH to obtain a clear solution. The organic layer was separated and the aqueous phase was extracted with CH_2_Cl_2_ (2 × 10 mL). The combined organic layers were dried over anhydrous Na_2_SO_4_, filtered and the solvent was removed in vacuo. The residual was purified by flash chromatography through a short silica column using petroleum ether as eluent.

**Isopropyl isothiocyanate** (CAS No: 2253-73-8, Table 2, entry 1): Yield: 85%; colorless oil; IR (neat): 2079 cm^−1^; ^1^H NMR (CDCl_3_) δ 1.37 (d, *J* = 7.0 Hz, 6H), 3.88–3.95 (m, 1H); GC–EIMS *m*/*z*: 101 [M^+^, 100%].

***n*-Butyl isothiocyanate** (CAS No: 592-82-5, Table 2, entry 2): Yield: 94%; colorless oil; IR (neat): 2099 cm^−1^; ^1^H NMR (CDCl_3_) δ 0.94–0.97 (t, *J* = 7.0 Hz, 3H), 1.42–1.50 (m, 2H), 1.66–1.72 (m, 2H), 3.53 (t, *J* = 6.5 Hz, 2H); GC–EIMS *m*/*z*: 115 [M^+^, 100%].

***tert*-Butyl isothiocyanate** (CAS No: 590-42-1, Table 2, entry 3): Yield: 80%; colorless oil; IR (neat): 2074 cm^−1^; ^1^H NMR (CDCl_3_) δ 1.44 (s, 9H); GC–EIMS *m*/*z*: 115 [M^+^, 100%].

***n*-Octyl isothiocyanate** (CAS No: 4430-45-9, Table 2, entry 4): Yield: 95%; colorless oil; IR (neat): 2102 cm^−1^; ^1^H NMR (CDCl_3_) δ 0.89 (t, *J* = 7.0 Hz, 3H), 1.28–1.32 (m, 8H), 1.39–1.42 (m, 2H), 1.67–1.72 (m, 2H), 3.51 (t, *J* = 7.0 Hz, 2H); GC–EIMS *m*/*z*: 171 [M^+^, 0%], 115 (100%).

**2-Ethyl-1-hexyl isothiocyanate** (CAS No: 21663-56-9, Table 2, entry 5): Yield: 98%; colorless oil; IR (neat): 2095 cm^−1^; ^1^H NMR (CDCl_3_) δ 0.90–0.93 (m, 6H), 1.25–1.44 (m, 8H), 1.56–1.61 (m, 1H), 3.47–3.49 (m, 2H); GC–EIMS *m*/*z*: 171 [M^+^, 4%], 57 (100%).

**Cyclohexyl isothiocyanate** (CAS No: 1122-82-3, Table 2, entry 6): Yield: 95%; colorless oil; IR (neat): 2103 cm^−1^; ^1^H NMR (CDCl_3_) δ 1.38–1.90 (m, 10H), 3.67–3.71 (m, 1H); GC–EIMS *m*/*z*: 141 [M^+^, 90%], 55 (100%).

**Benzyl isothiocyanate** (CAS No: 622-78-6, Table 2, entry 7): Yield: 99%; colorless oil; IR (neat): 2091 cm^−1^; ^1^H NMR (CDCl_3_) δ 4.71 (s, 2H), 7.31–7.41 (m, 5H); GC–EIMS *m*/*z*: 149 [M^+^, 30%], 91 (100%).

**1-Phenylethyl isothiocyanate** (CAS No: 4478-92-6, Table 2, entry 8): Yield: 99%; colorless oil; IR (neat): 2090 cm^−1^; ^1^H NMR (CDCl_3_) δ 1.67 (d, *J* = 6.5 Hz, 3H), 4.91 (q, *J* = 6.5 Hz, 1H), 7.31–7.40 (m, 5H); GC–EIMS *m*/*z*: 163 [M^+^, 6%], 105 (100%).

**Phenyl isothiocyanate** (CAS No: 103-72-0, Table 2, entry 9): Yield: 98%; colorless oil; IR (neat): 2079 cm^−1^; ^1^H NMR (CDCl_3_) δ 7.20–7.35 (m, 5H); GC–EIMS *m*/*z*: 135 [M^+^, 100%].

**4-Methoxyphenyl isothiocyanate** (CAS No: 2284-20-0, Table 2, entry 10): Yield: 86%; colorless oil; IR (neat): 2109 cm^−1^; ^1^H NMR (CDCl_3_) δ 3.81 (s, 3H), 6.84–7.18 (m, 4H); GC–EIMS *m*/*z*: 165 [M^+^, 100%].

**4-Methylphenyl isothiocyanate** (CAS No: 622-59-3, Table 2, entry 11): Yield: 93%; colorless oil; IR (neat): 2100 cm^−1^; ^1^H NMR (CDCl_3_) δ 2.34 (s, 3H), 7.10–7.15 (m, 4H); GC–EIMS *m*/*z*: 149 [M^+^, 100%].

**2-Methylphenyl isothiocyanate** (CAS No: 614-69-7, Table 2, entry 12): Yield: 95%; colorless oil; IR (neat): 2083 cm^−1^; ^1^H NMR (CDCl_3_) δ 2.38 (s, 3H), 7.16–7.22 (m, 4H); GC–EIMS *m*/*z*: 149 [M^+^, 100%].

**3-Methylphenyl isothiocyanate** (CAS No: 621-30-7, Table 2, entry 13): Yield: 98%; colorless oil; IR (neat): 2106 cm^−1^; ^1^H NMR (CDCl_3_) δ 2.34 (s, 3H), 7.02–7.24 (m, 4H); GC–EIMS *m*/*z*: 149 [M^+^, 100%].

#### Synthetic procedure for phenyl isothiocyanate in 1-mol scale

Into a 2-L jacketed flask, 91.2 g of CS_2_ (1.2 mol) was dropwise added to a mixture of aniline (93.0 g, 1.0 mol) and K_2_CO_3_ (276.0 g, 2.0 mol) in 700 mL of water at room temperature within a period of 2.5 h. After the addition was complete, the mixture was stirred another 2 h. Then, the mixture was cooled to 0 °C, and a solution of 92.3 g of TCT (0.5 mol) in 450 mL of CH_2_Cl_2_ was dropwise added within 4 h. After the addition was complete, the mixture was stirred another 1 h to complete the conversion. The resulting mixture was basified to pH >11 with 250 mL of 6 N NaOH and yielded a clear solution. The organic layer was separated and the aqueous layer was extracted with 150 mL of CH_2_Cl_2_. The combined organic layers were dried over anhydrous Na_2_SO_4_, filtered, and the solvent of filtrate was removed via distillation under atmospheric pressure with a 25-cm Vigreux column. The residue was vacuum distilled and the desired product fraction was collected at 72–74 °C/1 mmHg. Finally, 127.0 g of colorless liquid (94%) was obtained.

#### Typical procedure for the preparation of electron-deficient aryl isothiocyanates

A certain amount of CS_2_ and arylamine (20 mmol) was added to a solution of K_2_CO_3_ (5.52 g, 40 mmol) in 20 mL of water and 5 mL of DMF. The mixture was warmed to 40 °C for several hours to a constant conversion based on HPLC determination. The following operation was the same as the experimental procedure described above.

**4-Fluorophenyl isothiocyanate** (CAS No: 1544-68-9, Table 4, entry 1): Yield: 94%; colorless oil; IR (neat): 2079 cm^−1^; ^1^H NMR (CDCl_3_) δ 7.04–7.08 (m, 2H), 7.21–7.24 (m, 2H); GC–EIMS *m*/*z*: 153 [M^+^, 100%].

**4-Chlorophenyl isothiocyanate** (CAS No: 2131-55-7, Table 4, entry 2): Yield: 92%; white solid; mp 45–46 °C (lit. [57]: mp 44–45 °C); IR (KBr): 2037 cm^−1^; ^1^H NMR (CDCl_3_) δ 7.15–7.18 (m, 2H), 7.31–7.33 (m, 2H); GC–EIMS *m*/*z*: 169 [M^+^, 100%], 171 [M + 2, 44%].

**2-Chlorophenyl isothiocyanate** (CAS No: 2740-81-0, Table 4, entry 3): Yield: 90%; colorless oil; IR (neat): 2075 cm^−1^; ^1^H NMR (CDCl_3_) δ 7.19–7.25 (m, 3H), 7.41–7.43 (m, 1H); GC–EIMS *m*/*z*: 169 [M^+^, 100%], 171 [M + 2, 37%].

**3-Chlorophenyl isothiocyanate** (CAS No: 2392-68-9, Table 4, entry 4): Yield: 89%; colorless oil; IR (neat): 2097 cm^−1^; ^1^H NMR (CDCl_3_) δ 7.10–7.13 (m, 1H), 7.22–7.30 (m, 3H); GC–EIMS *m*/*z*: 169 [M^+^, 100%], 171 [M + 2, 34%].

**4-Trifluoromethylphenyl isothiocyanate** (CAS No: 1645-65-4, Table 4, entry 5): Yield: 89%; White solid; mp 40–41 °C (lit. [52]: mp 43 °C); IR (KBr): 2091 cm^−1^; ^1^H NMR (CDCl_3_) δ 7.31–7.34 (m, 2H), 7.62–7.63 (m, 2H); GC–EIMS *m*/*z*: 203 [M^+^, 100%].

**4-Acetophenyl isothiocyanate** (CAS No: 2131-57-9, Table 4, entry 6): Yield: 86%; white solid; mp 73–74 °C (lit. [63]: mp 75–76 °C); IR (KBr): 2125, 1680 cm^−1^; ^1^H NMR (CDCl_3_) δ 2.61 (s, 3H), 7.30–7.32 (m, 2H), 7.96–7.98 (m, 2H); GC–EIMS *m*/*z*: 177 [M^+^, 70%], 162 (100%).

**4-Cyanophenyl isothiocyanate** (CAS No: 2719-32-6, Table 4, entry 7): Yield: 91%; white solid; mp 121–122 °C (lit. [63]: mp 119–120 °C); IR (KBr): 2138 cm^−1^; ^1^H NMR (CDCl_3_) δ 7.30–7.32 (m, 2H), 7.65–7.67 (m, 2H); GC–EIMS *m*/*z*: 160 [M^+^, 100%].

**4-Nitrophenyl isothiocyanate** (CAS No: 2131-61-5, Table 4, entry 8): Yield: 13%; white solid; mp 107–108 °C (lit. [63]: mp 108–110 °C); IR (KBr): 2120 cm^−1^; ^1^H NMR (CDCl_3_) δ 7.36–7.38 (m, 2H), 8.26–8.27 (m, 2H); GC–EIMS *m*/*z*: 180 [M^+^, 100%].

**1,4-Diisothiocyanatobenzene** (CAS No: 4044-65-9, Table 4, entry 8): Yield: 50%; white solid; mp 129–130 °C (lit. [64]: mp 132 °C); IR (KBr): 2072 cm^−1^; ^1^H NMR (CDCl_3_) δ 7.22 (s, 4H); GC–EIMS *m*/*z*: 192 [M^+^, 100%].

## Supporting Information

File 1 Proton NMR and GC–MS spectra.

## References

[1] Fahey J W, Zalcmann A T, Talalay P (2001). Phytochemistry.

[2] Nakamura Y, Miyoshi N (2010). Biosci, Biotechnol, Biochem.

[3] Pedras M S C, Zheng Q-A, Gadagi R S (2007). Chem Commun.

[4] Munday R, Zhang Y, Munday C M, Bapardekar M V, Paonessa J D (2008). Pharm Res.

[5] Tian X, Huters A D, Douglas C J, Garg N K (2009). Org Lett.

[6] Adsule S, Banerjee S, Ahmed F, Padhye S, Sarkar F H (2010). Bioorg Med Chem Lett.

[7] Mukerjee A K, Ashare R (1991). Chem Rev.

[8] Sommen G (2004). Synlett.

[9] Li J, Tan Z, Tang S, Hewlett I, Pang R, He M, He S, Tian B, Chen K, Yang M (2009). Bioorg Med Chem.

[10] Kang I-J, Wang L-W, Yeh T-K, Lee C-C, Lee Y-C, Hsu S-J, Wu Y-S, Wang J-C, Chao Y-S, Yueh A (2010). Bioorg Med Chem.

[11] Peng H, Liang Y, Chen L, Fu L, Wang H, He H (2011). Bioorg Med Chem Lett.

[12] Doyle A G, Jacobsen E N (2007). Chem Rev.

[13] Toshimitsu A, Uemura S, Okano M, Watanabe N (1983). J Org Chem.

[14] Albanese D, Penso M (1991). Synthesis.

[15] Shan W G, Bian G F, Su W K, Liang X R (2004). Org Prep Proced Int.

[16] Kim J N, Ryu E K (1993). Tetrahedron Lett.

[17] Kim J N, Jung K S, Lee H J, Son J S (1997). Tetrahedron Lett.

[18] Adam W, Bargon R M, Bosio S G, Schenk W A, Stalke D (2002). J Org Chem.

[19] Arisawa M, Ashikawa M, Suwa A, Yamaguchi M (2005). Tetrahedron Lett.

[20] Valette L, Poulain S, Fernandez X, Lizzani-Cuvelier L (2005). J Sulfur Chem.

[21] Isoda T, Hayashi K, Tamai S, Kumagai T, Nagao Y (2006). Chem Pharm Bull.

[22] Zhong B, Al-Awar R S, Shih C, Grimes J H, Vieth M, Hamdouchi C (2006). Tetrahedron Lett.

[23] Neely W J (1960). Aust J Chem.

[24] Bollini M, Casal J J, Alvarez D E, Boiani L, González M, Cerecetto H, Bruno A M (2009). Bioorg Med Chem.

[25] Dyer E, Johnson T B (1932). J Am Chem Soc.

[26] Dyson G M, Harrington T (1942). J Chem Soc.

[27] Jochims J C, Seeliger A (1965). Tetrahedron.

[28] Gottfried R (1966). Angew Chem, Int Ed Engl.

[29] Larsen C, Steliou K, Harpp D N (1978). J Org Chem.

[30] Larsen C, Harpp D N (1981). J Org Chem.

[31] Kim S, Yi K Y (1986). J Org Chem.

[32] Kim S, Yi K Y (1985). Tetrahedron Lett.

[33] Grayson J I (1997). Org Process Res Dev.

[34] Hodgkins J E, Ettlinger M G (1956). J Org Chem.

[35] Hodgkins J E, Reeves W P (1964). J Org Chem.

[36] Shibanuma T, Shiono M, Mukaiyama T (1977). Chem Lett.

[37] Kondo K, Komamura C, Murakami M, Takemoto K (1985). Synth Commun.

[38] Yamamoto T, Terada A, Muramatsu T, Ikeda K (1994). Org Prep Proced Int.

[39] Boas U, Jakobsen M H (1995). J Chem Soc, Chem Commun.

[40] Boas U, Pedersen B, Christensen J B (1998). Synth Commun.

[41] Boas U, Gertz H, Christensen J B, Heegaard P M H (2004). Tetrahedron Lett.

[42] Li G, Tajima H, Ohtani T (1997). J Org Chem.

[43] Isobe T (1999). J Org Chem.

[44] Bian G, Shan W, Su W (2005). J Chem Res.

[45] Chaskar A C, Yewale S, Bhagat R, Langi B P (2008). Synth Commun.

[46] Wong R, Dolman S J (2007). J Org Chem.

[47] Bian G, Qiu H, Jiang J, Wu J, Lai G (2007). Phosphorus, Sulfur Silicon Relat Elem.

[48] Ghosh H, Yella R, Nath J, Patel B K (2008). Eur J Org Chem.

[49] Munch H, Hansen J S, Pittelkow M, Christensen J B, Boas U (2008). Tetrahedron Lett.

[50] Chaskar A C, Bandgar B P, Modhave R K, Patil A B, Yewale S (2009). Synth Commun.

[51] Nath J, Ghosh H, Yella R, Patel B K (2009). Eur J Org Chem.

[52] Jamir L, Ali A R, Ghosh H, Chipem F A S, Patel B K (2010). Org Biomol Chem.

[53] Yella R, Ghosh H, Murru S, Sahoo S K, Patel B K (2010). Synth Commun.

[54] Dains F B, Brewster R G, Olander C P (1941). Organic Syntheses.

[55] Moore M L, Crossley F S (1955). Organic Syntheses.

[56] Li Z, Qian X, Liu Z, Li Z, Song G (2000). Org Prep Proced Int.

[57] van der Kerk G J M, Pluygers C W, de Vries G (1973). Organic Syntheses.

[58] Heusenstamm G G, Schreyer G, Vanheertum R (1978). U.S. Patent.

[59] Mesheram H M, Dale S, Yadav J S (1997). Tetrahedron Lett.

[60] Furumoto S (1971). Nippon Kagaku Zasshi.

[61] Henke K R, Hutchison A R, Krepps M K, Parkin S, Atwood D A (2001). Inorg Chem.

[62] Xiao W-J, Alper H (1999). J Org Chem.

[63] Sayigh A A R, Ulrich H, Potts J S (1965). J Org Chem.

[64] Lieber E, Slutkin R (1962). J Org Chem.

