# Supplementary material for: A general and facile one-pot process of isothiocyanates from amines under aqueous conditions
Source: Beilstein J Org Chem. 2012 Jan 10;8:61–70. doi: 10.3762/bjoc.8.6 (PMC3302099; doi:10.3762/bjoc.8.6)
Supplement: File 1 — Proton NMR and GC–MS spectra. [file Beilstein_J_Org_Chem-08-61-s001.pdf]

Supporting Information

for

A general and facile one-pot process of isothiocyanates from amines under aqueous conditions

Nan Sun[1], Bin Li[1], Jianping Shao[1], Weimin Mo* [1], Baoxiang Hu[1], Zhenlu Shen[1] and Xinquan Hu*[1,2]

Address: [1]College of Chemical Engineering and Materials Science, Zhejiang University of Technology, Hangzhou 310014, China and [2]State Key Laboratory for Oxo Synthesis and Selective Oxidation, Lanzhou Institute of Chemical Physics, Chinese Academy of Sciences, Lanzhou 730000, China

E-mail: Weimin Mo - mowm@zjut.edu.cn; Xinquan Hu - xinquan@zjut.edu.cn

* Corresponding author

Proton NMR and GC–MS spectra

Proton NMR and GC-MS spectra for isothiocyanates

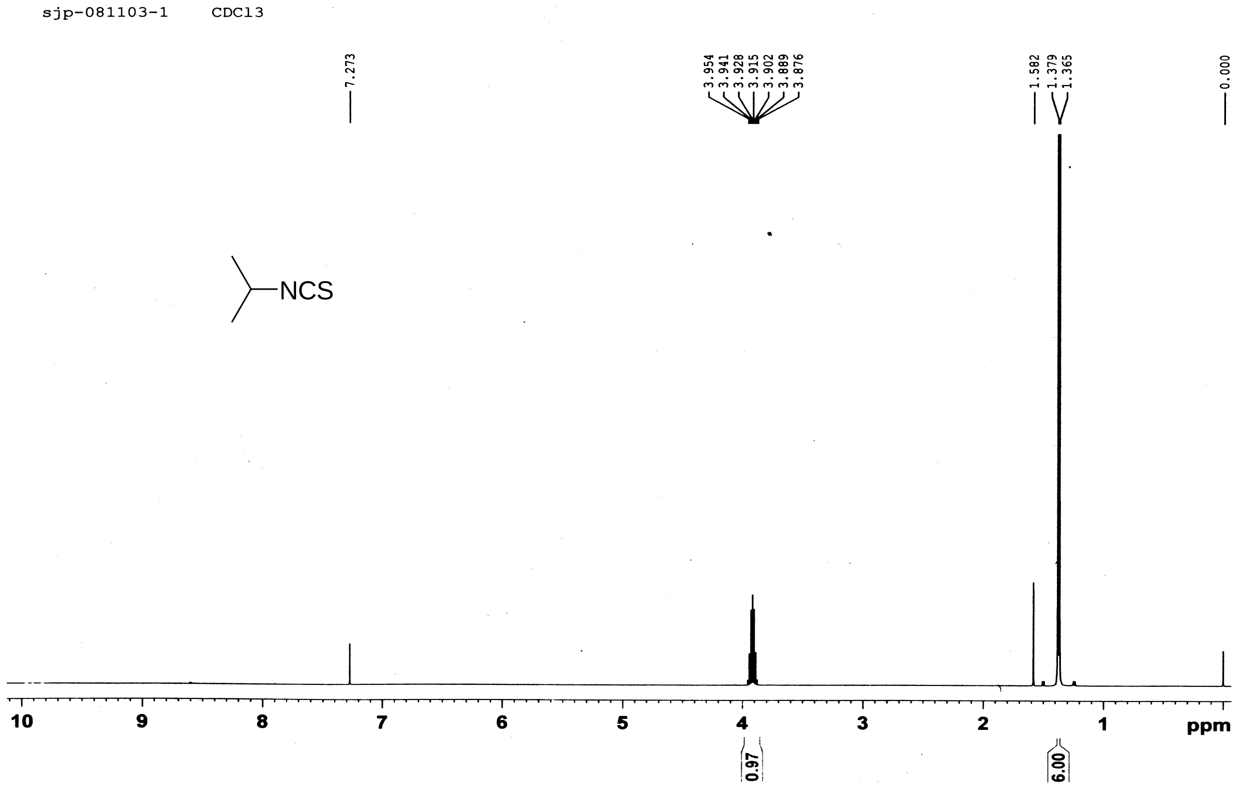

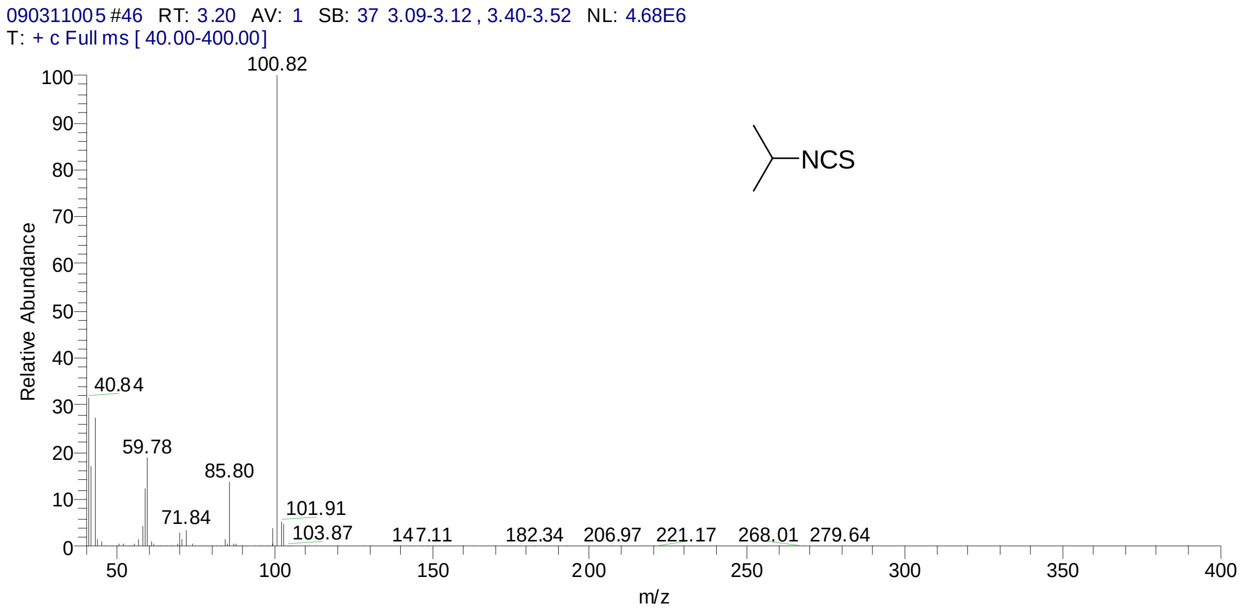

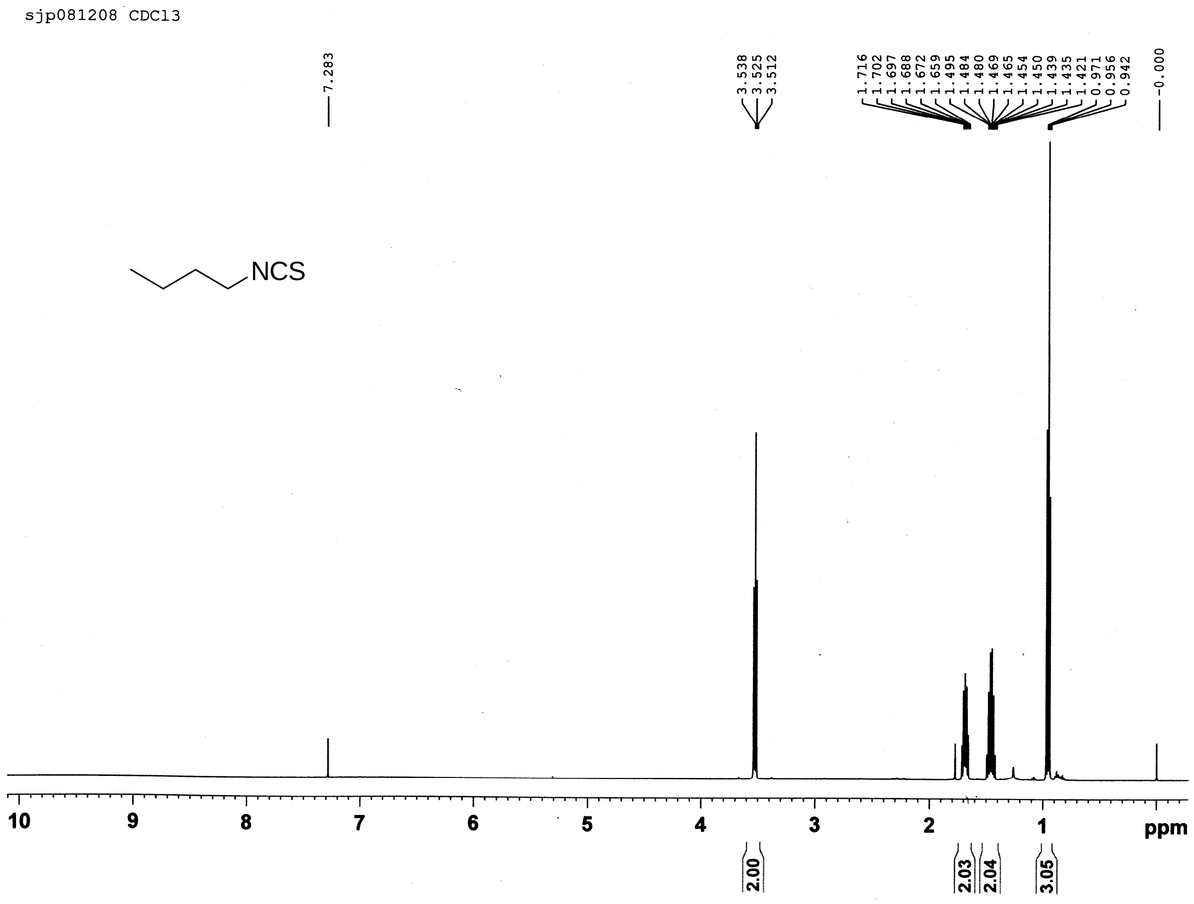

sjp081208 CDCl3
7.283
3.538
3.525
3.512
0.000
NCS
ppm
2.00
2.03
2.04
3.05

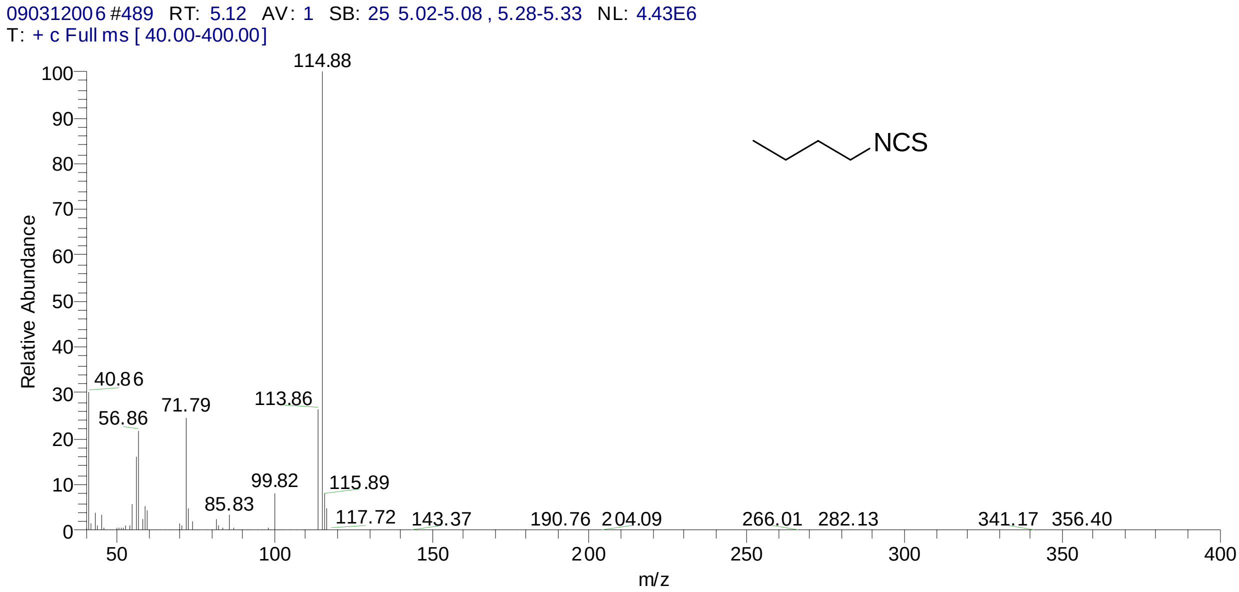

090312006 #489 RT: 5.12 AV: 1 SB: 25 5.02-5.08 , 5.28-5.33 NL: 4.43E6
T: + c Full ms [40.00-400.00]
114.88
NCS
Relative Abundance
40.86
56.86
71.79
113.86
99.82
115.89
85.83
117.72
143.37
190.76
204.09
266.01
282.13
341.17
356.40
m/z

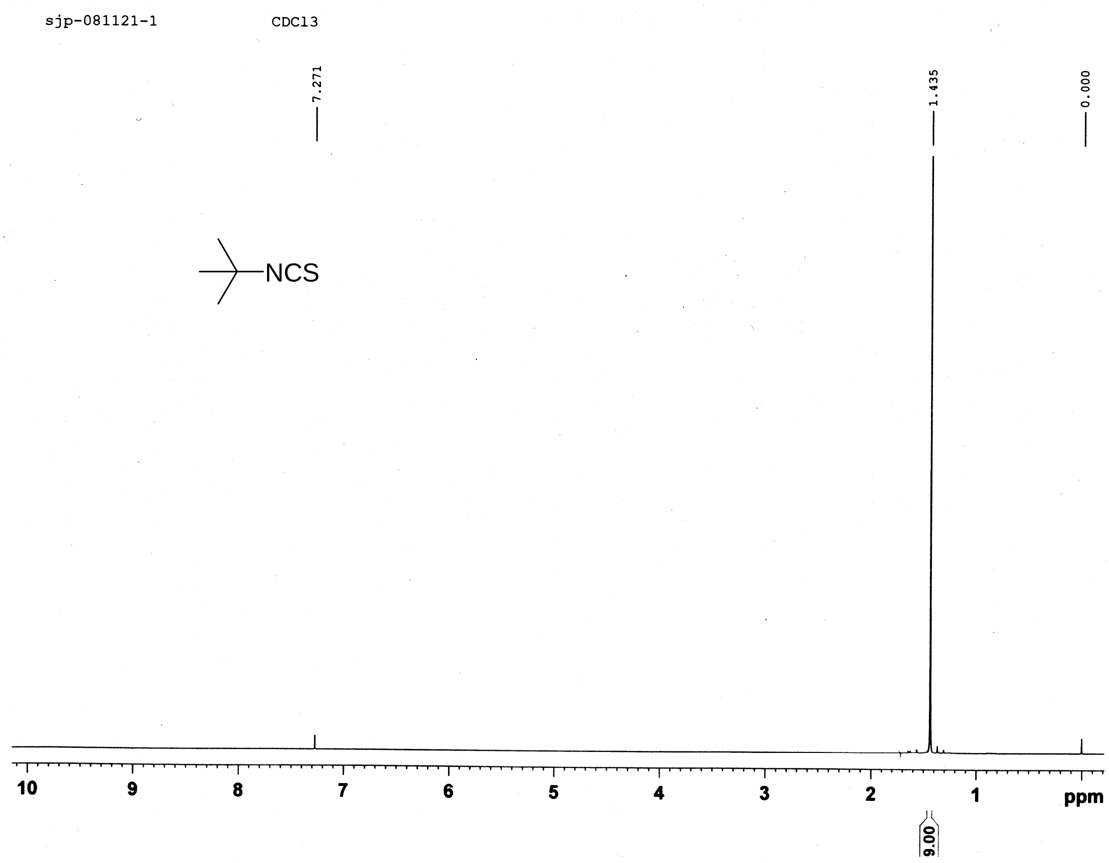
sjp-081121-1
CDCl3
7.271
1.435
0.000
NCS
10
9
8
7
6
5
4
3
2
1
ppm
9.00

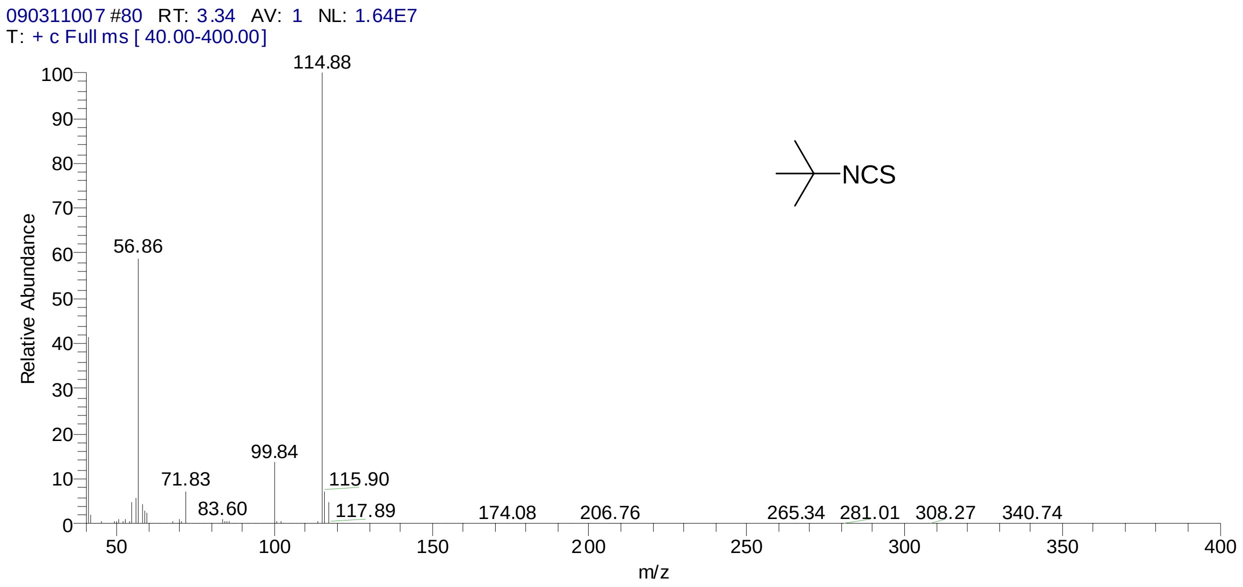
0903110 07 #80 RT: 3.34 AV: 1 NL: 1.64E7
T: + c Full ms [40.00-400.00]
114.88
56.86
99.84
71.83
83.60
115.90
117.89
174.08
206.76
265.34
281.01
308.27
340.74
NCS
Relative Abundance
m/z

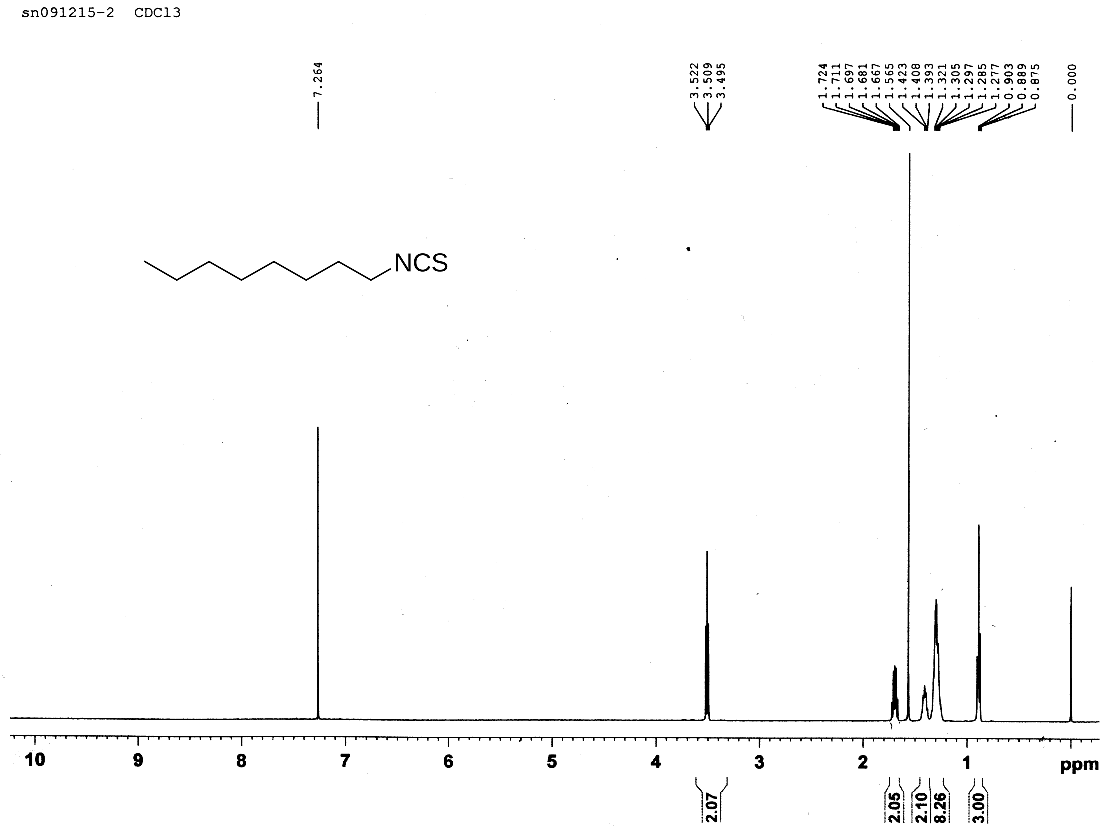
sn091215-2 CDCl3
7.264
3.522
3.509
3.495
1.724
1.711
1.697
1.681
1.667
1.565
1.423
1.408
1.393
1.321
1.305
1.297
1.285
1.277
0.903
0.889
0.875
0.000
NCS
10 9 8 7 6 5 4 3 2 1 ppm
2.07
2.05
2.10
8.26
3.00

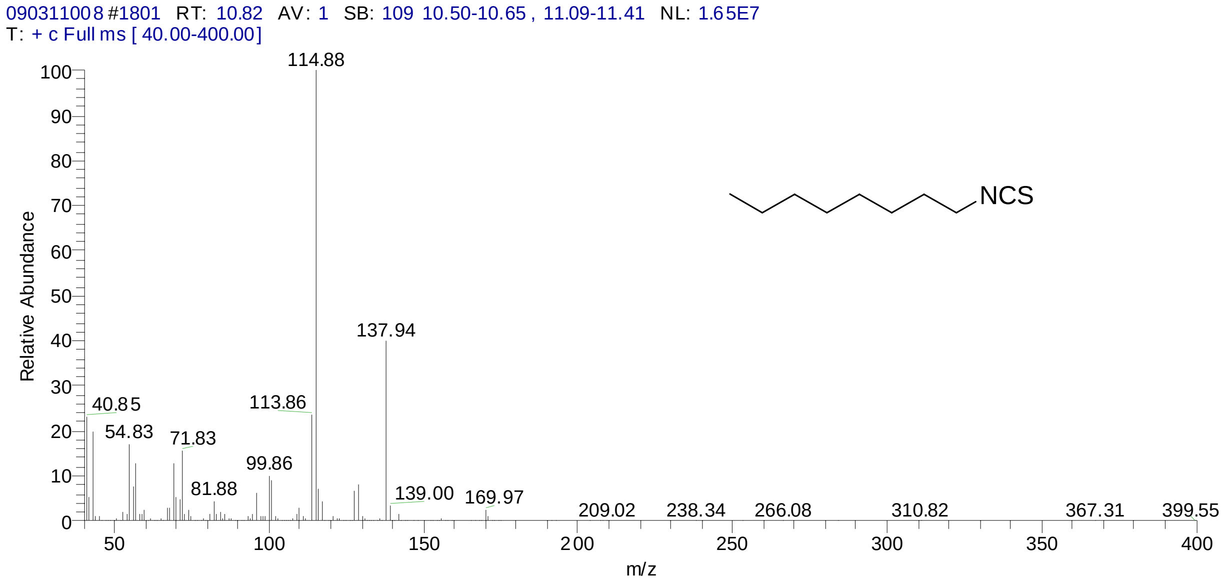
090311008 #1801 RT: 10.82 AV: 1 SB: 109 10.50-10.65 , 11.09-11.41 NL: 1.65E7
T: + c Full ms [40.00-400.00]
114.88
137.94
40.85
54.83
71.83
113.86
99.86
81.88
139.00
169.97
209.02
238.34
266.08
310.82
367.31
399.55
NCS
Relative Abundance
m/z

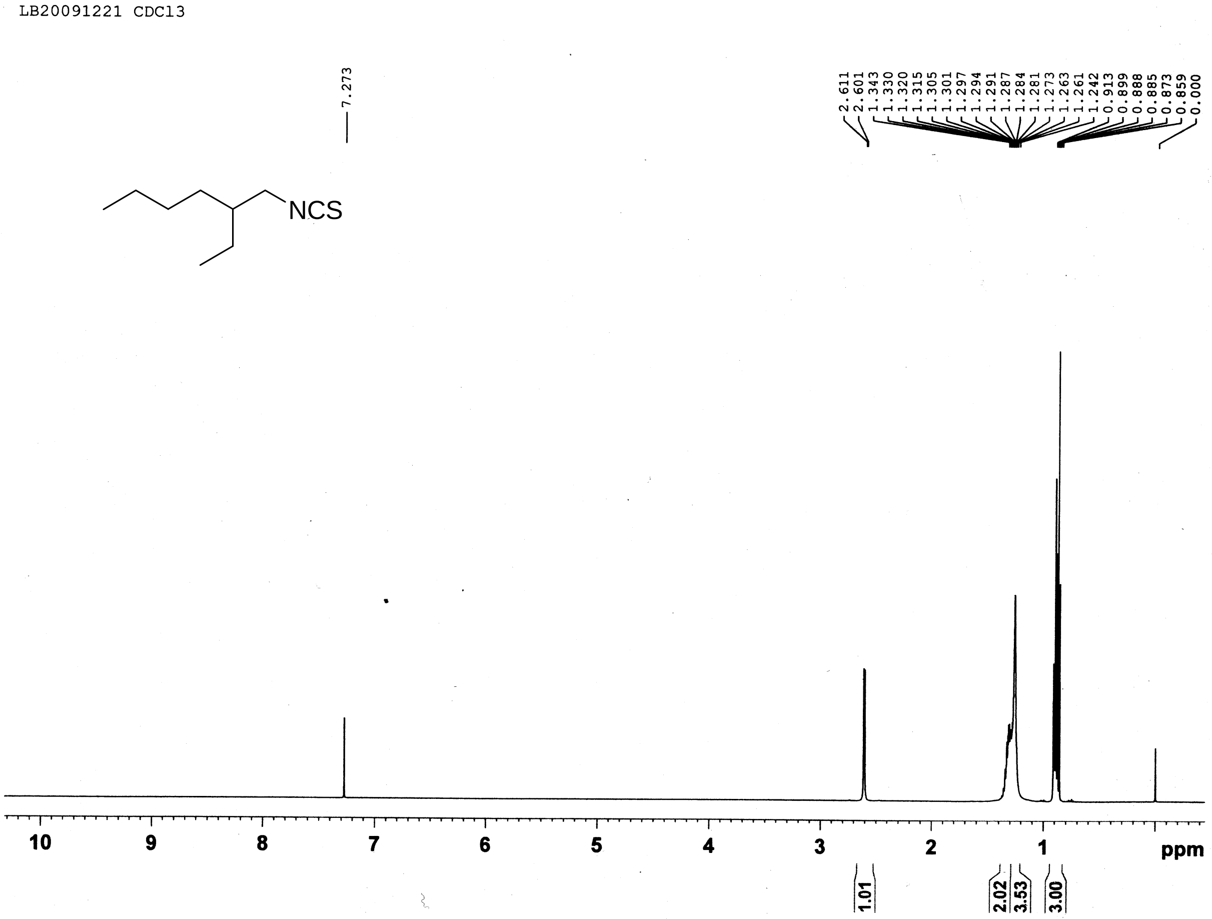

LB20091221 CDCl3
7.273
2.611
2.601
1.343
1.330
1.320
1.315
1.305
1.301
1.297
1.294
1.291
1.287
1.284
1.281
1.273
1.263
1.261
1.242
0.913
0.899
0.888
0.885
0.873
0.859
0.000
NCS
10 9 8 7 6 5 4 3 2 1 ppm
1.01
2.02
3.53
3.00

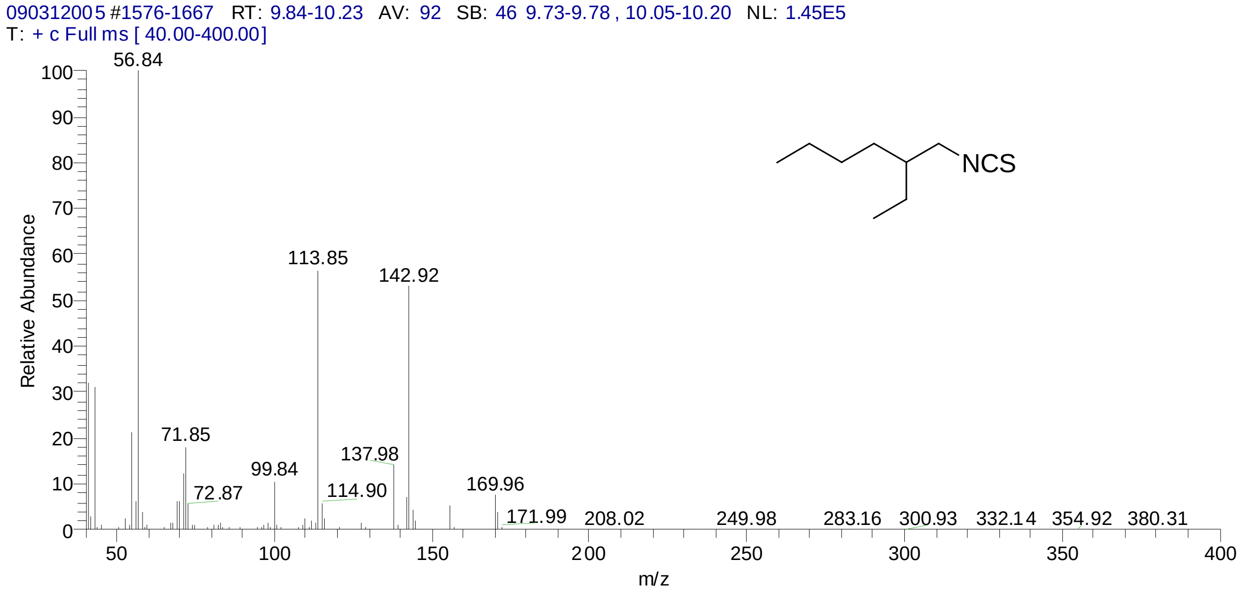

090312005 #1576-1667 RT: 9.84-10.23 AV: 92 SB: 46 9.73-9.78 , 10.05-10.20 NL: 1.45E5
T: + c Full ms [40.00-400.00]
56.84
113.85
142.92
71.85
72.87
99.84
137.98
114.90
169.96
171.99
208.02
249.98
283.16
300.93
332.14
354.92
380.31
Relative Abundance
m/z
NCS

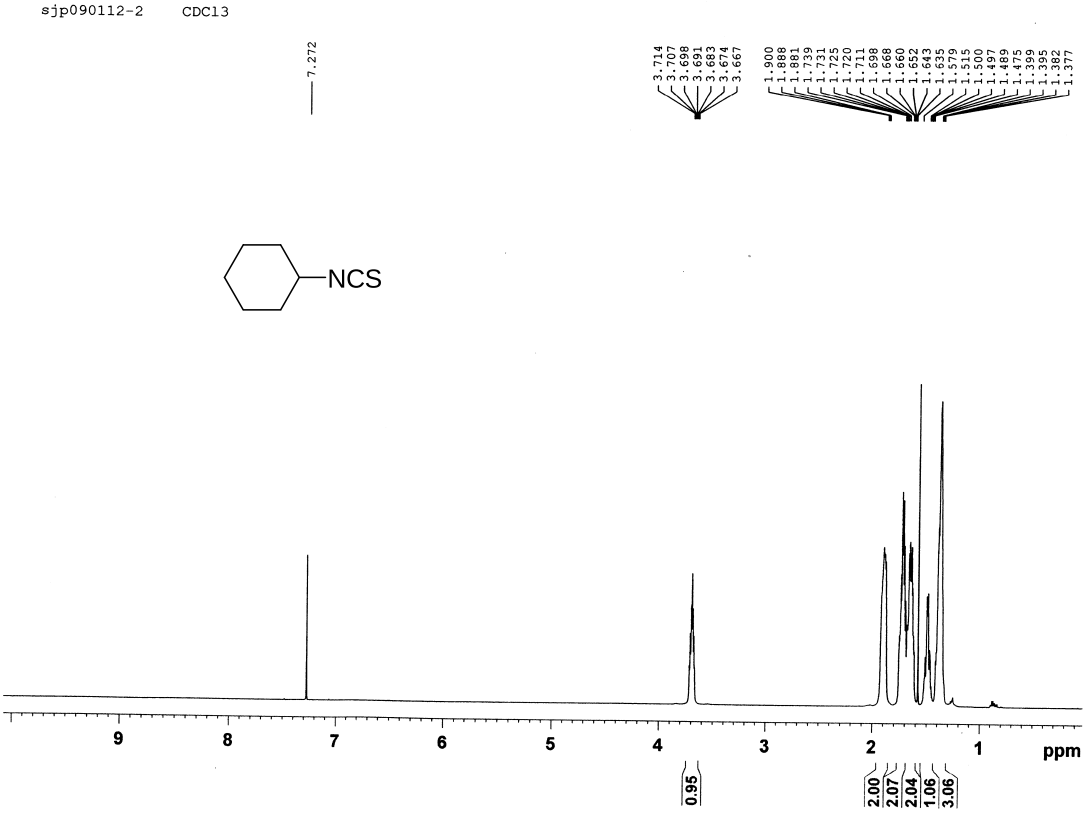

sjp090112-2 CDCl3
7.272
3.714 3.707 3.698 3.691 3.683 3.674 3.667
1.900 1.888 1.881 1.739 1.731 1.725 1.720 1.711 1.698 1.668 1.660 1.652 1.643 1.635 1.579 1.515 1.500 1.497 1.489 1.475 1.399 1.395 1.382 1.377
NCS
9 8 7 6 5 4 3 2 1 ppm
0.95
2.00 2.07 2.04 1.06 3.06

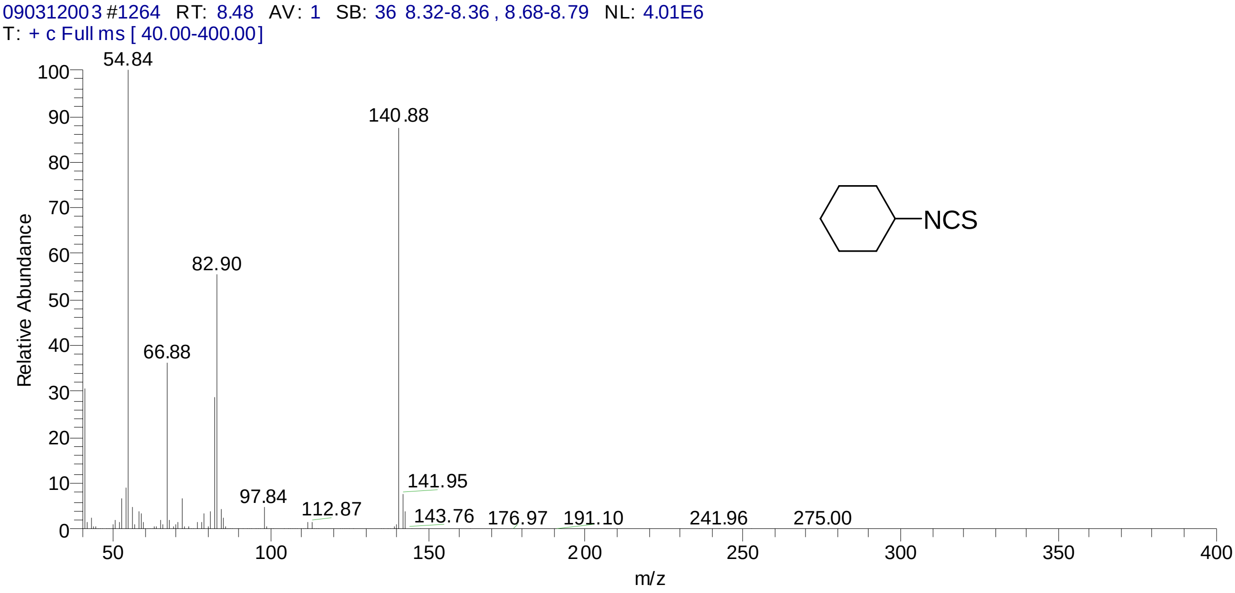

090312003 #1264 RT: 8.48 AV: 1 SB: 36 8.32-8.36 , 8.68-8.79 NL: 4.01E6
T: + c Full ms [40.00-400.00]
Relative Abundance
54.84
140.88
82.90
66.88
97.84
112.87
141.95
143.76
176.97
191.10
241.96
275.00
NCS
m/z

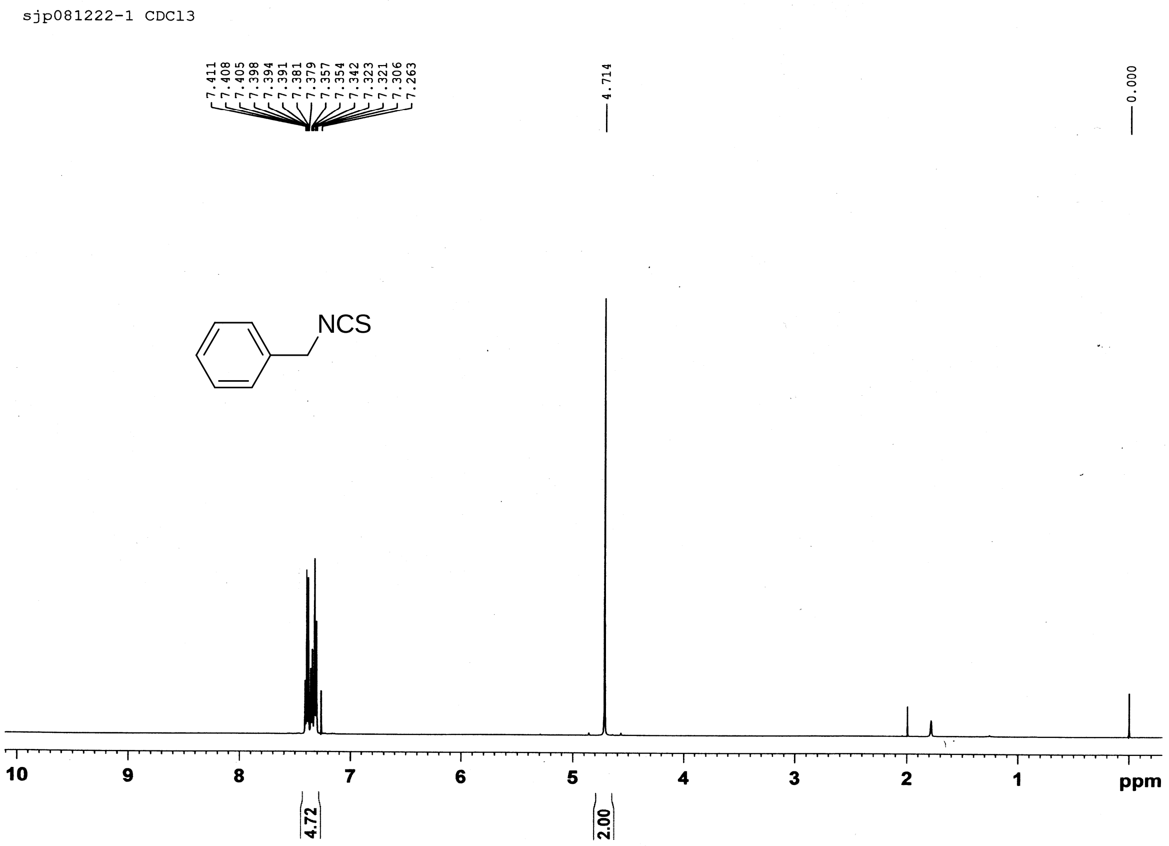

sjp081222-1 CDCl3
7.411
7.408
7.405
7.398
7.394
7.391
7.381
7.379
7.357
7.354
7.342
7.323
7.321
7.306
7.263
4.714
0.000
NCS
10
9
8
7
6
5
4
3
2
1
ppm
4.72
2.00

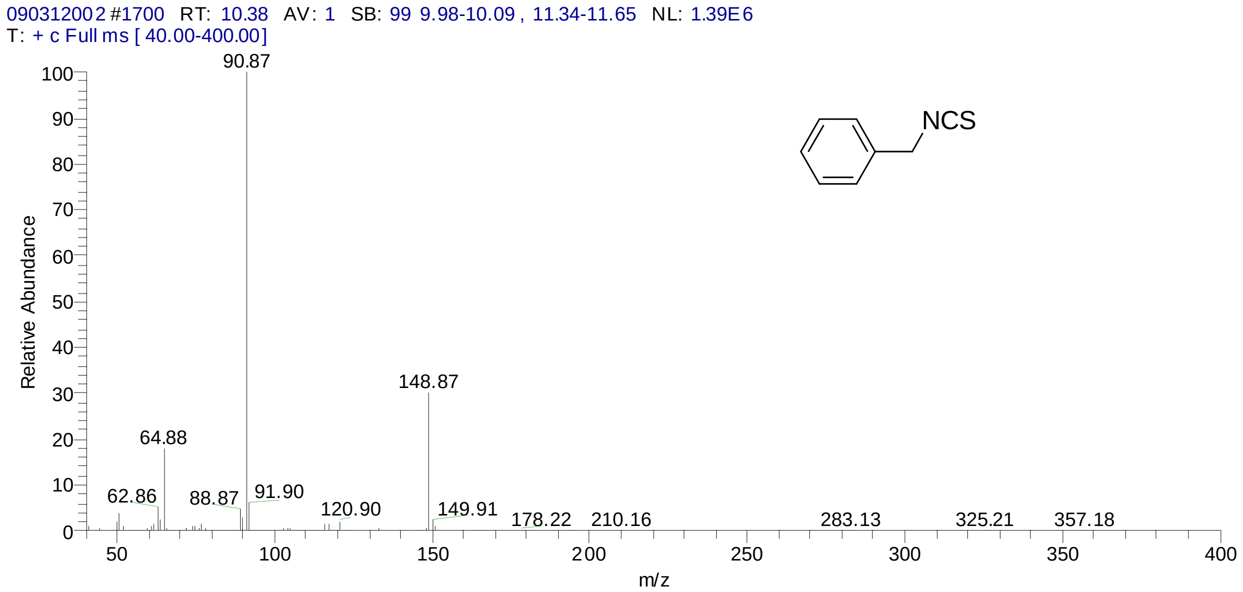

090312002 #1700 RT: 10.38 AV: 1 SB: 99 9.98-10.09 , 11.34-11.65 NL: 1.39E6
T: + c Full ms [40.00-400.00]
90.87
148.87
64.88
62.86
88.87
91.90
120.90
149.91
178.22
210.16
283.13
325.21
357.18
Relative Abundance
m/z
NCS

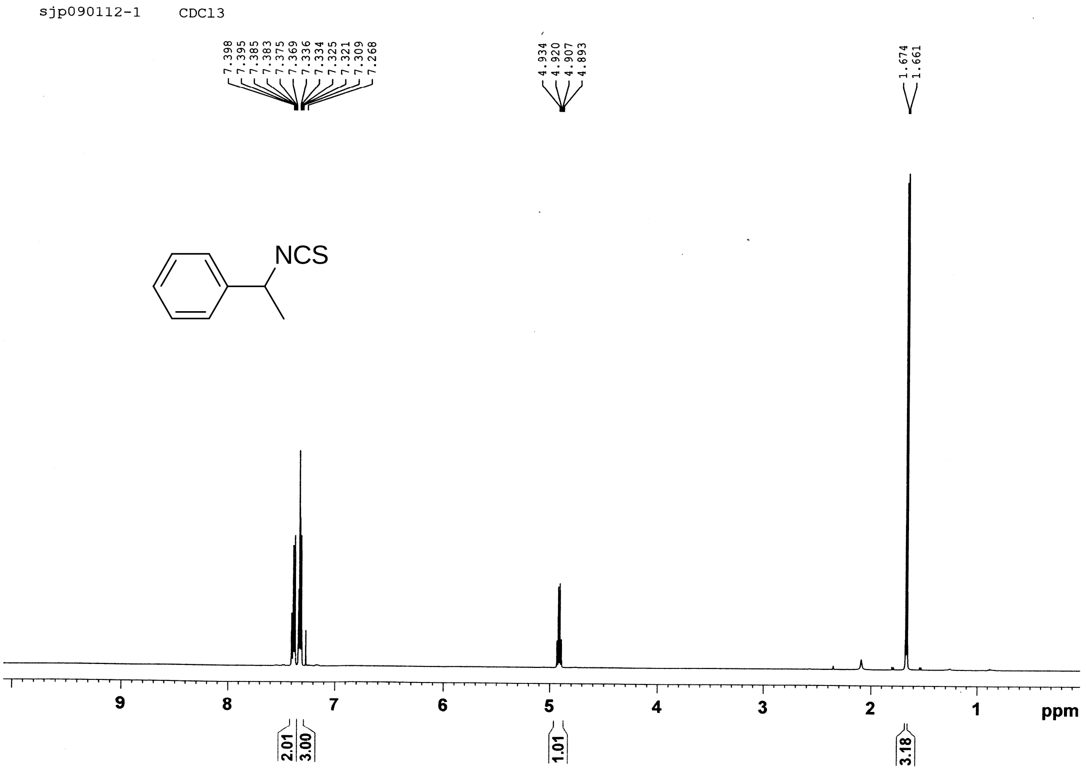

sjp090112-1 CDCl3
7.398
7.395
7.385
7.383
7.375
7.369
7.336
7.334
7.325
7.321
7.309
7.268
4.934
4.920
4.907
4.893
1.674
1.661
NCS
9
8
7
6
5
4
3
2
1
ppm
2.01
3.00
1.01
3.18

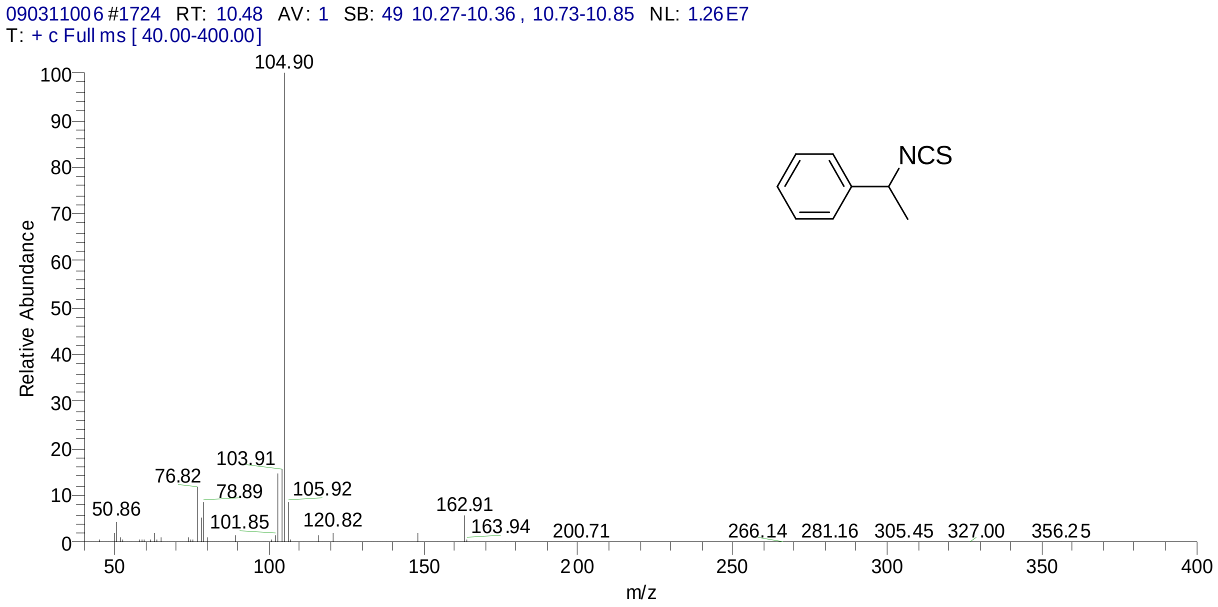

090311006 #1724 RT: 10.48 AV: 1 SB: 49 10.27-10.36 , 10.73-10.85 NL: 1.26E7
T: + c Full ms [40.00-400.00]
104.90
NCS
Relative Abundance
103.91
76.82
78.89
105.92
50.86
101.85
120.82
162.91
163.94
200.71
266.14
281.16
305.45
327.00
356.25
m/z

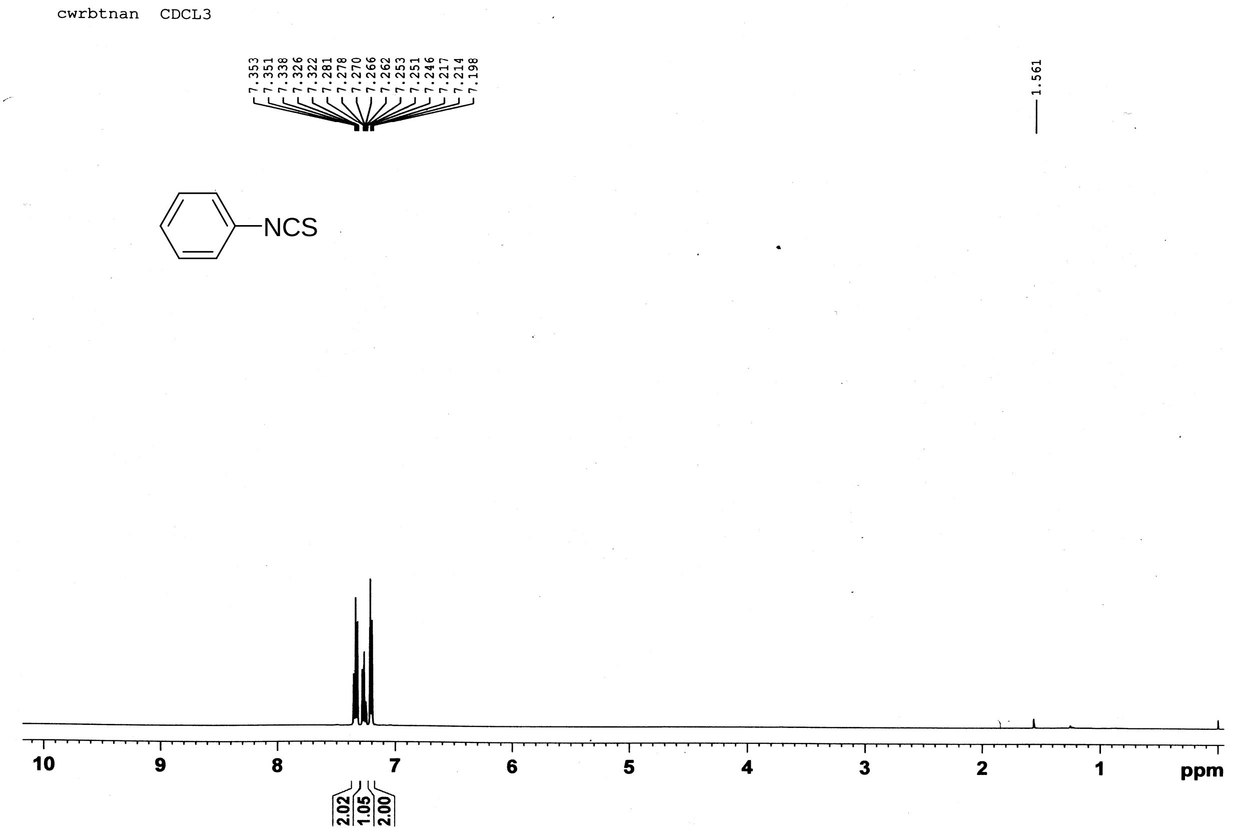
cwrbtnan CDCL3
7.353
7.351
7.338
7.326
7.322
7.281
7.278
7.270
7.266
7.262
7.253
7.251
7.246
7.217
7.214
7.198
1.561
NCS
10
9
8
7
6
5
4
3
2
1
ppm
2.02
1.05
2.00

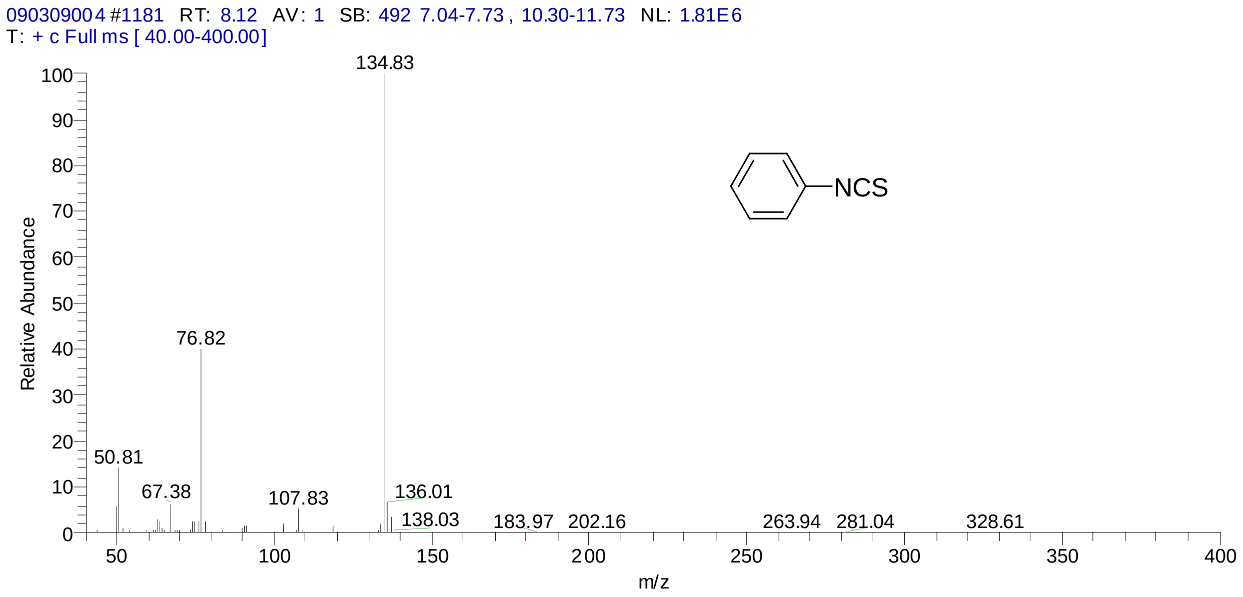
090309004 #1181 RT: 8.12 AV: 1 SB: 492 7.04-7.73 , 10.30-11.73 NL: 1.81E6
T: + c Full ms [40.00-400.00]
134.83
76.82
50.81
67.38
107.83
136.01
138.03
183.97
202.16
263.94
281.04
328.61
NCS
Relative Abundance
m/z

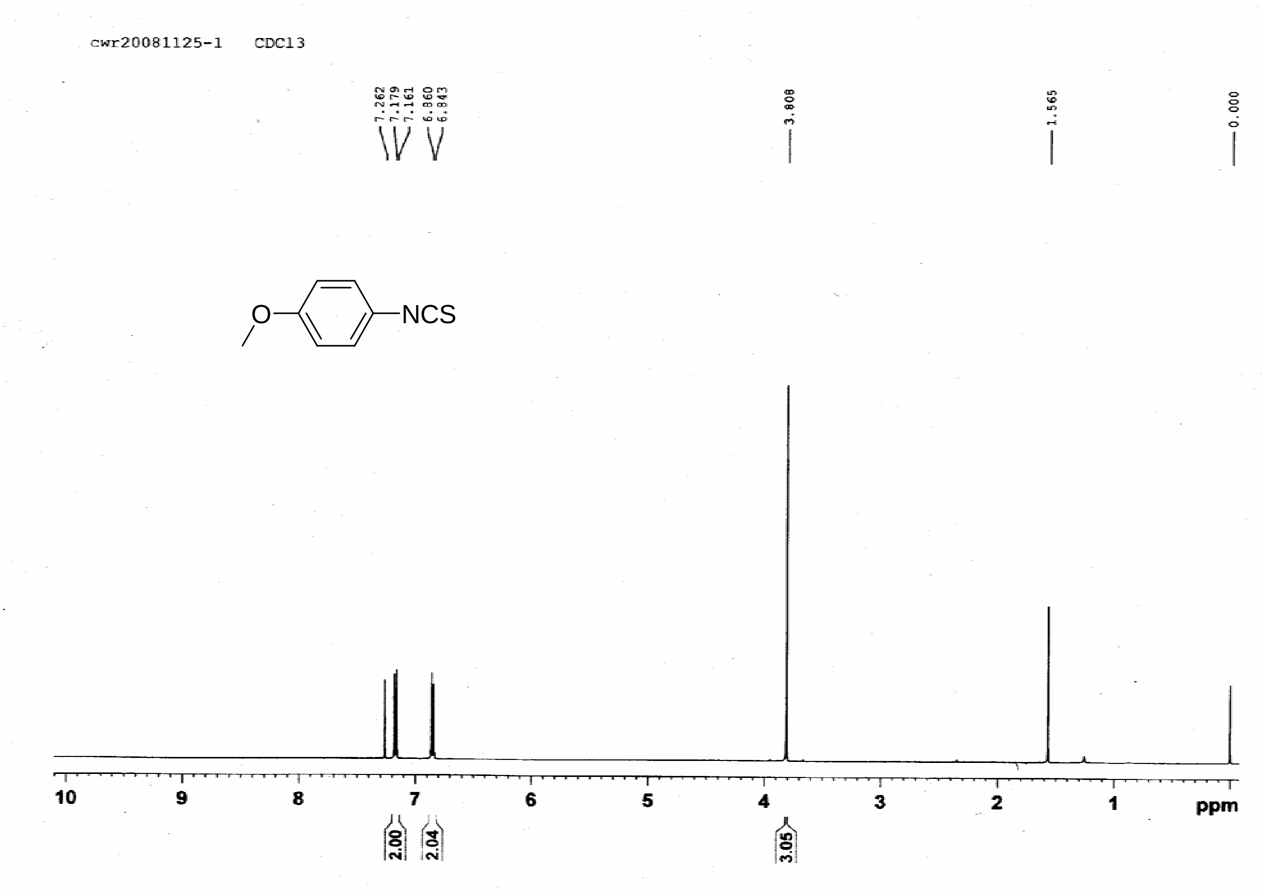
cwr20081125-1 CDCl3
7.262
7.179
7.161
6.860
6.843
3.808
1.565
0.000
NCS
O
10
9
8
7
6
5
4
3
2
1
ppm
2.00
2.04
3.05

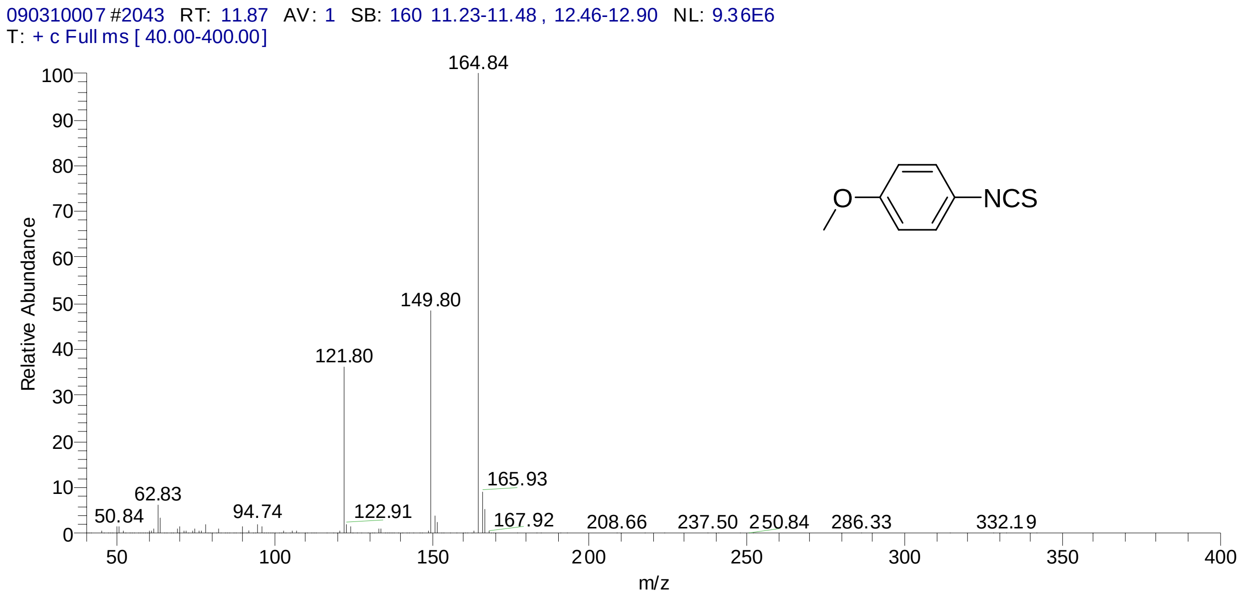
090310007 #2043 RT: 11.87 AV: 1 SB: 160 11.23-11.48 , 12.46-12.90 NL: 9.36E6
T: + c Full ms [40.00-400.00]
164.84
149.80
121.80
165.93
62.83
50.84
94.74
122.91
167.92
208.66
237.50
250.84
286.33
332.19
Relative Abundance
m/z
NCS
O

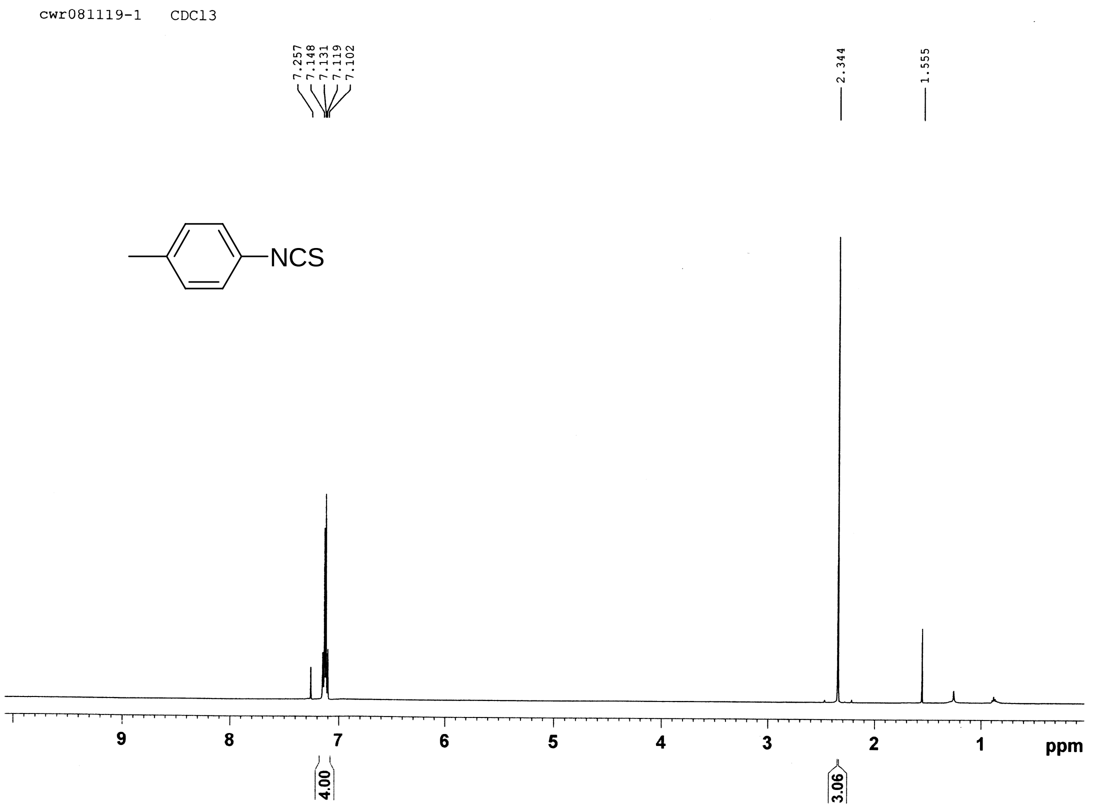
cwr081119-1 CDCl3
7.257
7.148
7.131
7.119
7.102
2.344
1.555
NCS
9
8
7
6
5
4
3
2
1
ppm
4.00
3.06

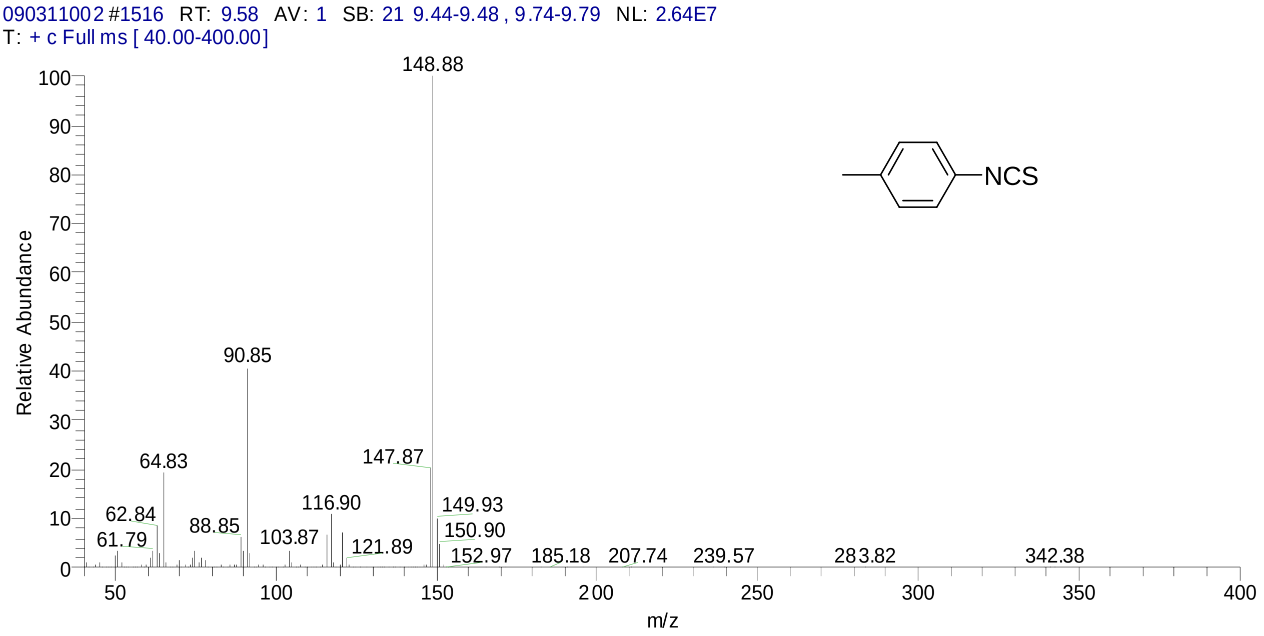
090311002 #1516 RT: 9.58 AV: 1 SB: 21 9.44-9.48 , 9.74-9.79 NL: 2.64E7
T: + c Full ms [40.00-400.00]
Relative Abundance
148.88
90.85
64.83
147.87
62.84
116.90
149.93
88.85
150.90
61.79
103.87
121.89
152.97
185.18
207.74
239.57
283.82
342.38
NCS
m/z

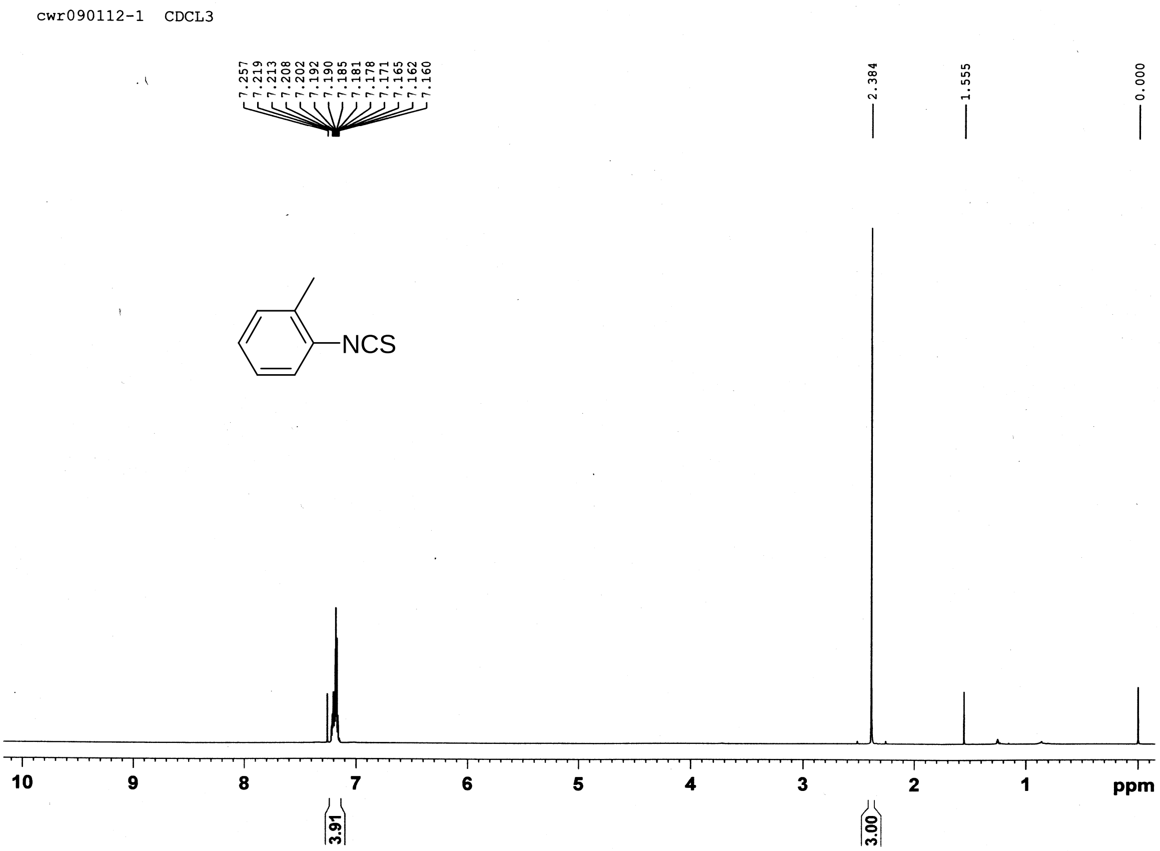
cwr090112-1 CDCL3
7.257
7.219
7.213
7.208
7.202
7.192
7.190
7.185
7.181
7.178
7.171
7.165
7.162
7.160
2.384
1.555
0.000
NCS
10
9
8
7
6
5
4
3
2
1
ppm
3.91
3.00

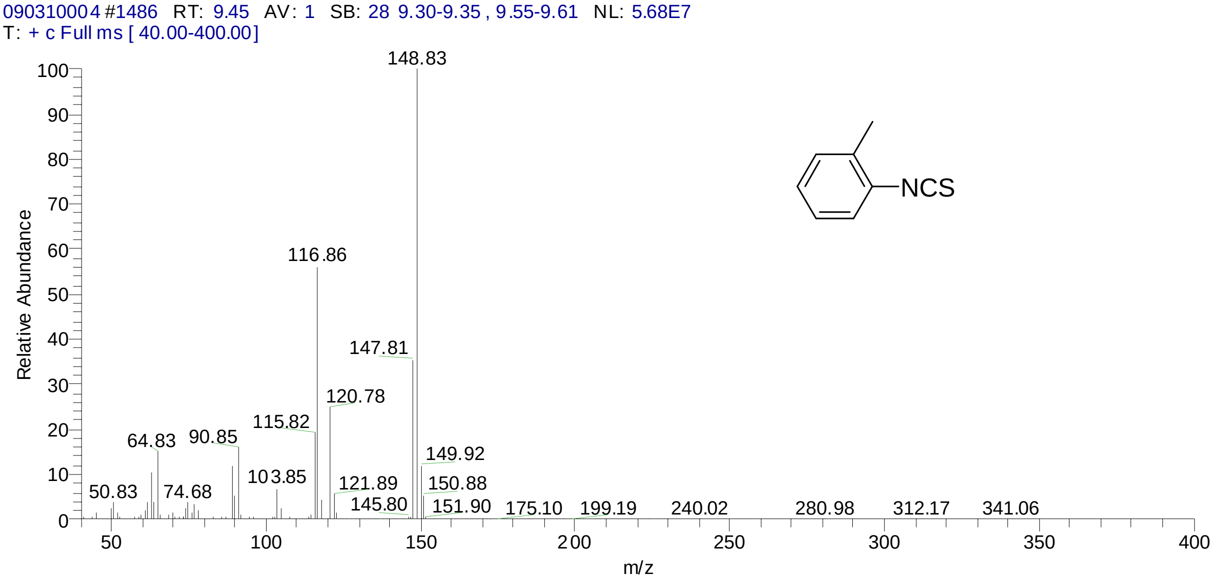
090310004 #1486 RT: 9.45 AV: 1 SB: 28 9.30-9.35 , 9.55-9.61 NL: 5.68E7
T: + c Full ms [40.00-400.00]
Relative Abundance
m/z
148.83
116.86
147.81
120.78
115.82
64.83
90.85
149.92
150.88
103.85
121.89
50.83
74.68
145.80
151.90
175.10
199.19
240.02
280.98
312.17
341.06
NCS

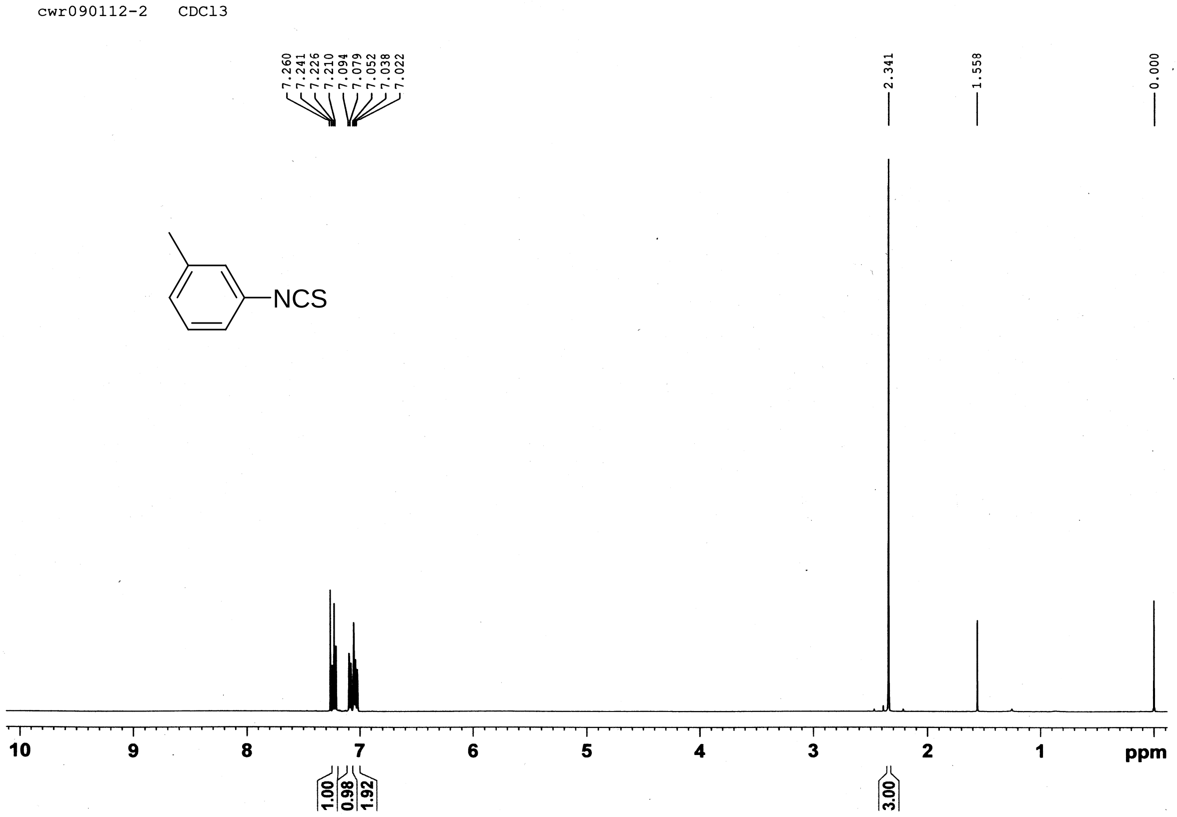

cwr090112-2 CDCl3
7.260
7.241
7.226
7.210
7.094
7.079
7.052
7.038
7.022
2.341
1.558
0.000
NCS
10
9
8
7
6
5
4
3
2
1
ppm
1.00
0.98
1.92
3.00

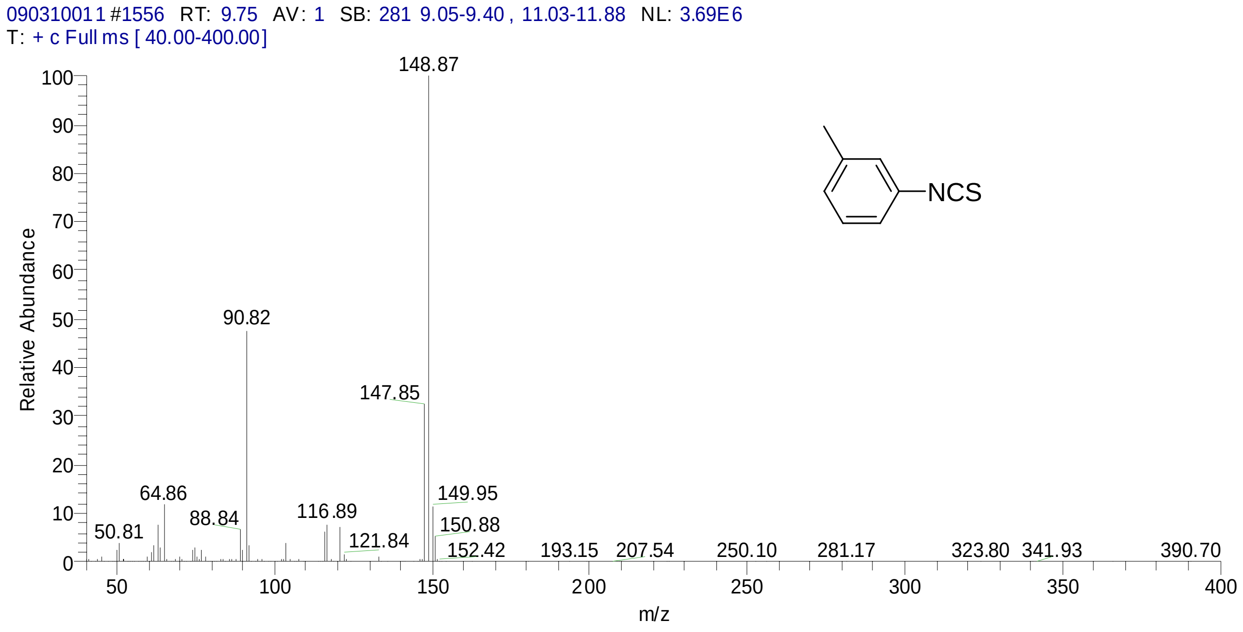

090310011 #1556 RT: 9.75 AV: 1 SB: 281 9.05-9.40 , 11.03-11.88 NL: 3.69E6
T: + c Full ms [40.00-400.00]
Relative Abundance
m/z
148.87
90.82
147.85
64.86
88.84
50.81
116.89
121.84
149.95
150.88
152.42
193.15
207.54
250.10
281.17
323.80
341.93
390.70
NCS

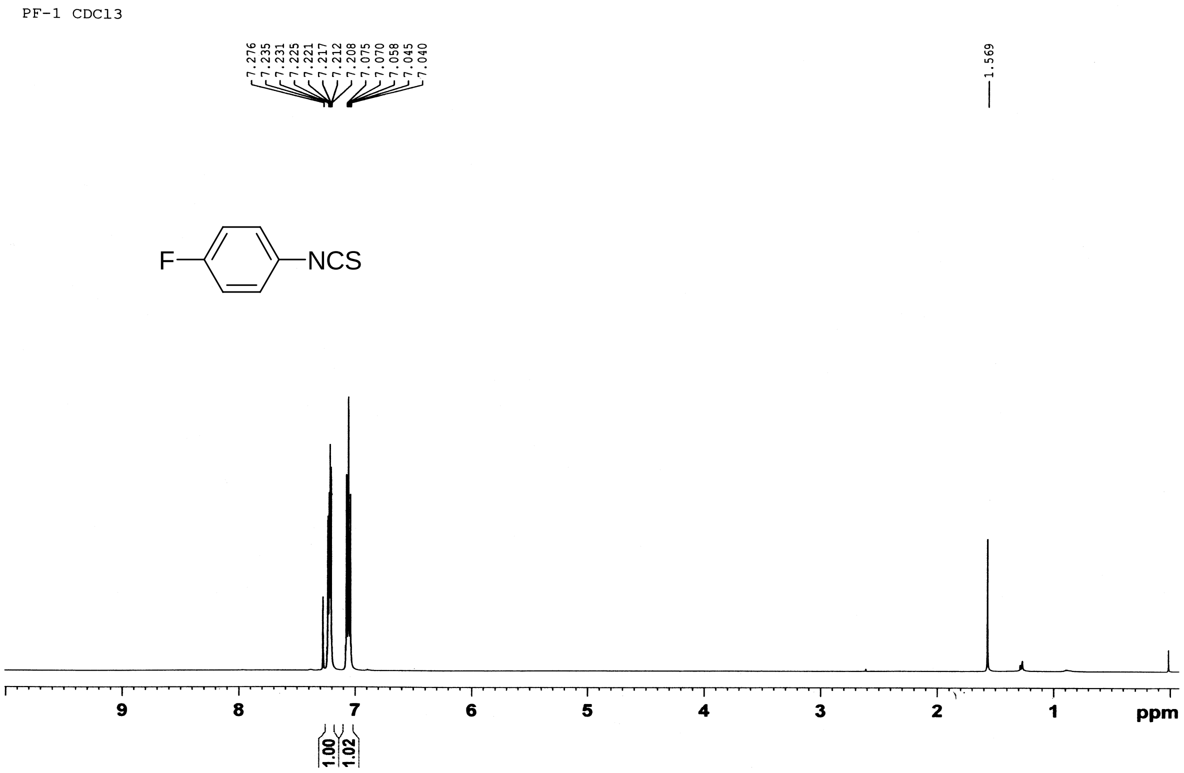
PF-1 CDCl3
7.276
7.235
7.231
7.225
7.221
7.217
7.212
7.208
7.075
7.070
7.058
7.045
7.040
1.569
F
NCS
9
8
7
6
5
4
3
2
1
ppm
1.00
1.02

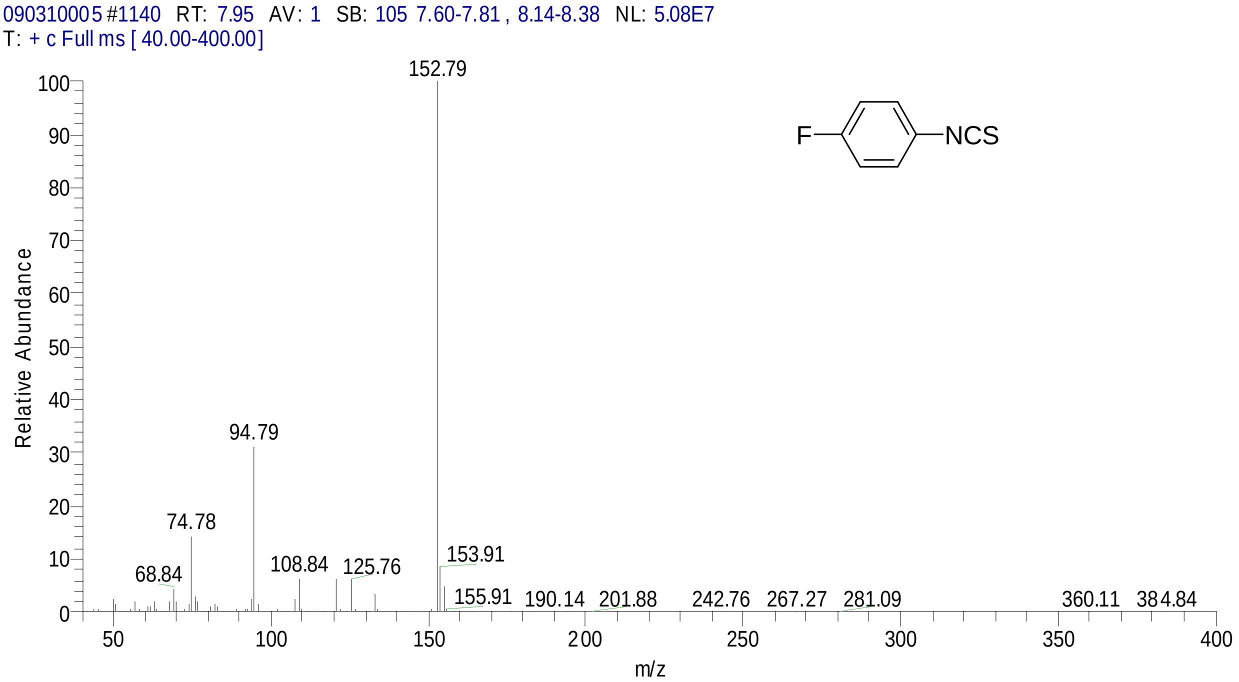
090310005 #1140 RT: 7.95 AV: 1 SB: 105 7.60-7.81 , 8.14-8.38 NL: 5.08E7
T: + c Full ms [40.00-400.00]
152.79
F
NCS
Relative Abundance
94.79
74.78
68.84
108.84
125.76
153.91
155.91
190.14
201.88
242.76
267.27
281.09
360.11
384.84
m/z

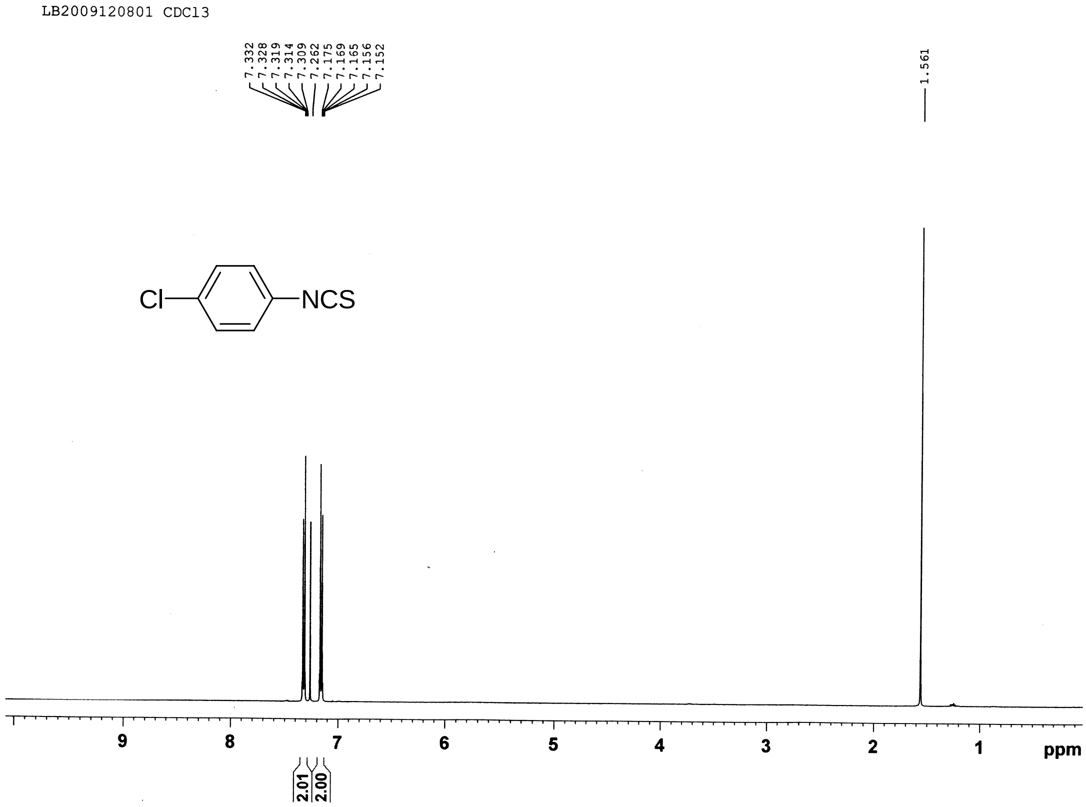
LB2009120801 CDCl3
7.332
7.328
7.319
7.314
7.309
7.262
7.175
7.169
7.165
7.156
7.152
1.561
Cl
NCS
9
8
7
6
5
4
3
2
1
ppm
2.01
2.00

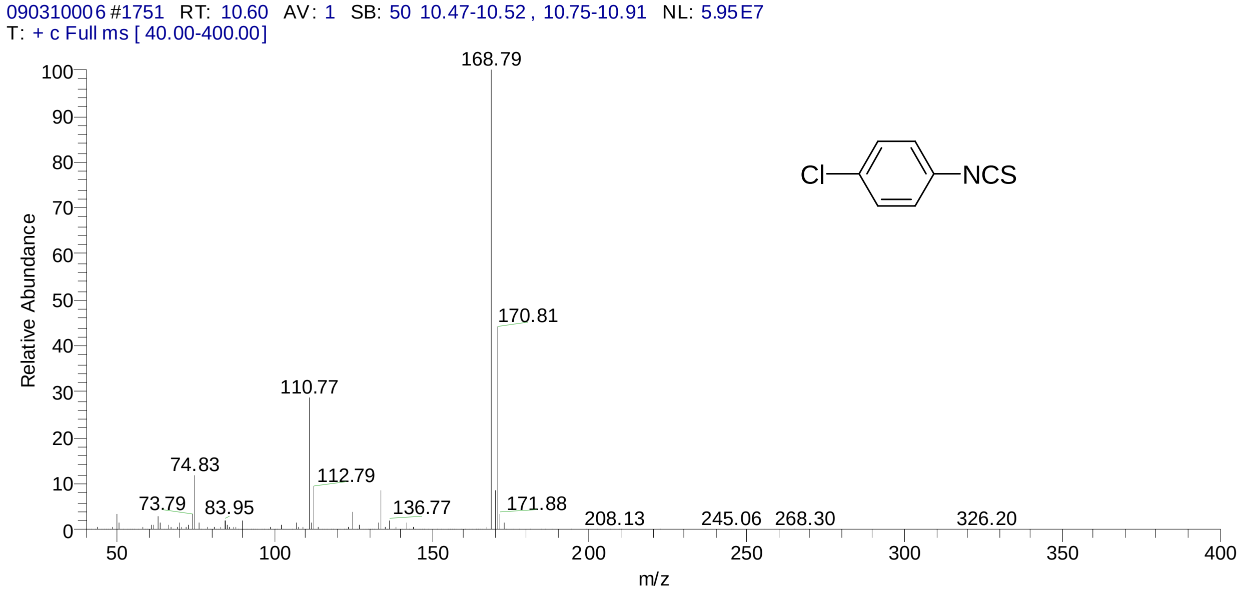
090310006 #1751 RT: 10.60 AV: 1 SB: 50 10.47-10.52 , 10.75-10.91 NL: 5.95E7
T: + c Full ms [40.00-400.00]
Relative Abundance
168.79
170.81
110.77
74.83
112.79
73.79
83.95
136.77
171.88
208.13
245.06
268.30
326.20
Cl
NCS
m/z

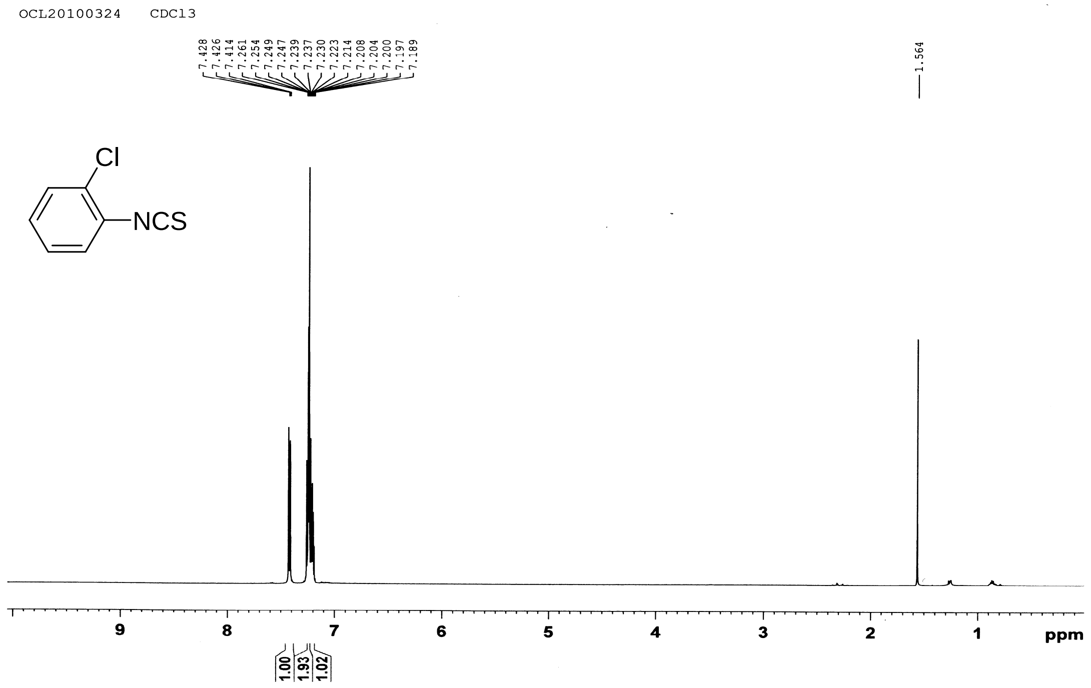

OCL20100324 CDCl3
7.428
7.426
7.414
7.261
7.254
7.249
7.247
7.239
7.237
7.230
7.223
7.214
7.208
7.204
7.200
7.197
7.189
1.564
Cl
NCS
9
8
7
6
5
4
3
2
1
ppm
1.00
1.93
1.02

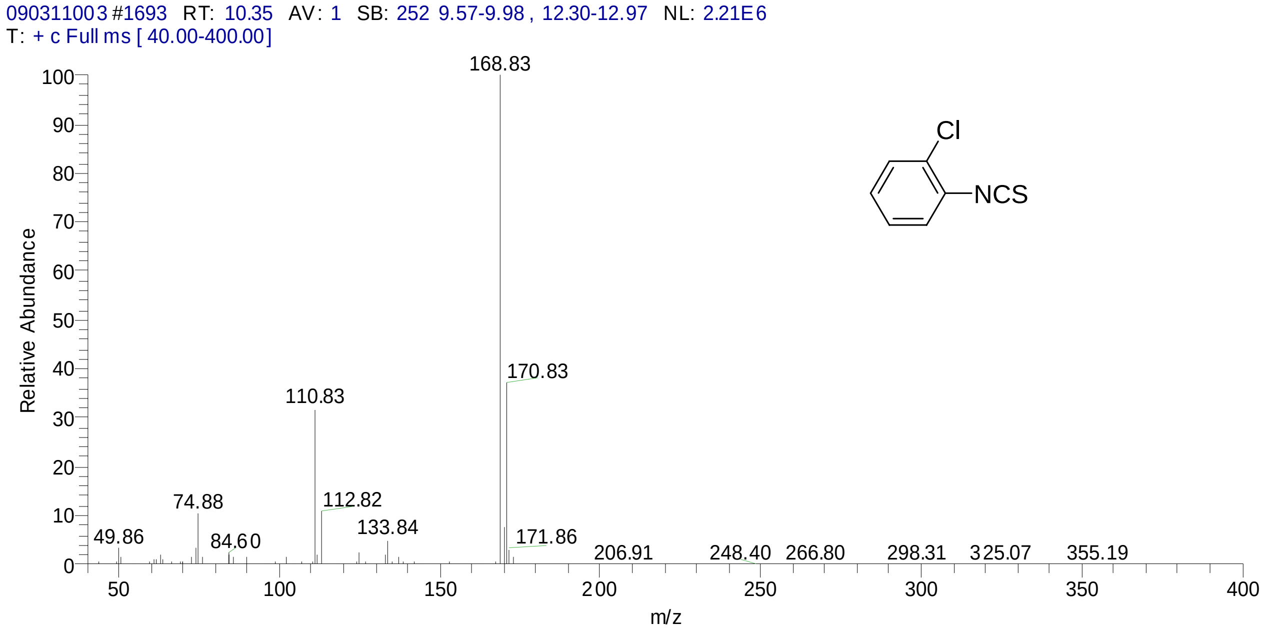

090311003 #1693 RT: 10.35 AV: 1 SB: 252 9.57-9.98 , 12.30-12.97 NL: 2.21E6
T: + c Full ms [40.00-400.00]
168.83
Cl
NCS
Relative Abundance
170.83
110.83
74.88
112.82
133.84
49.86
84.60
171.86
206.91
248.40
266.80
298.31
325.07
355.19
m/z

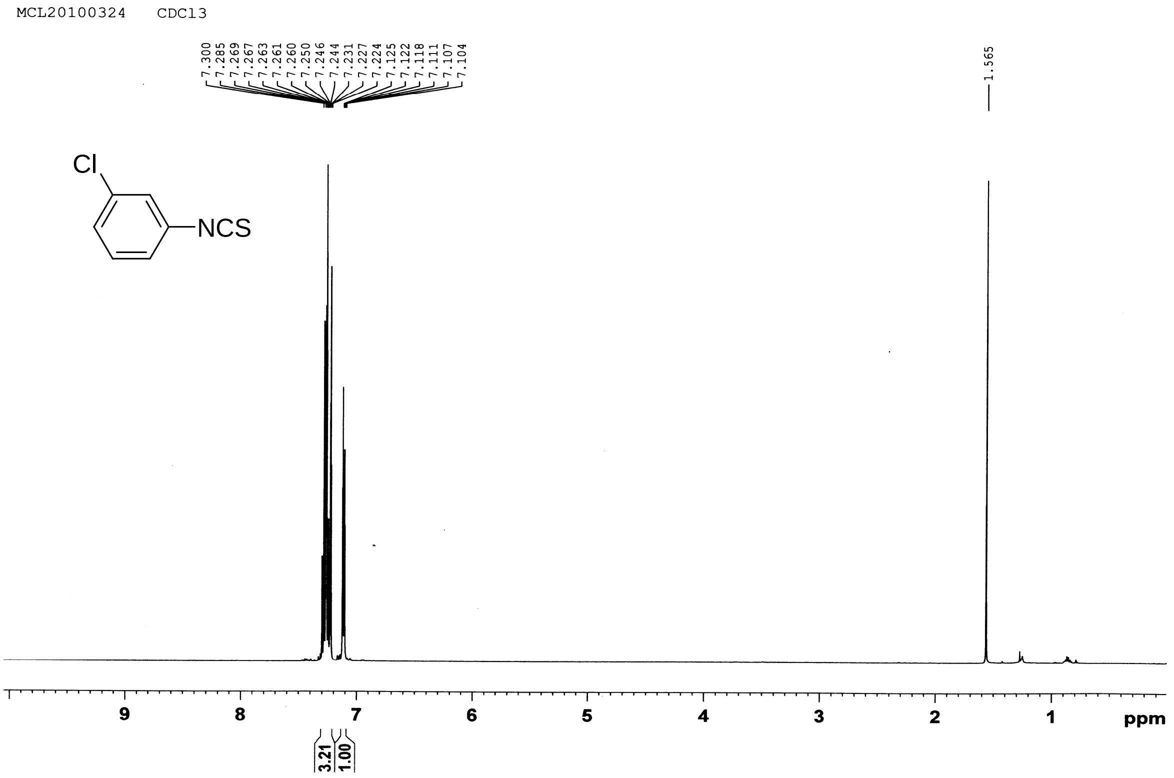
MCL20100324 CDCl3
7.300
7.285
7.269
7.267
7.263
7.261
7.260
7.250
7.246
7.244
7.231
7.227
7.224
7.125
7.122
7.118
7.111
7.107
7.104
1.565
Cl
NCS
9
8
7
6
5
4
3
2
1
ppm
3.21
1.00

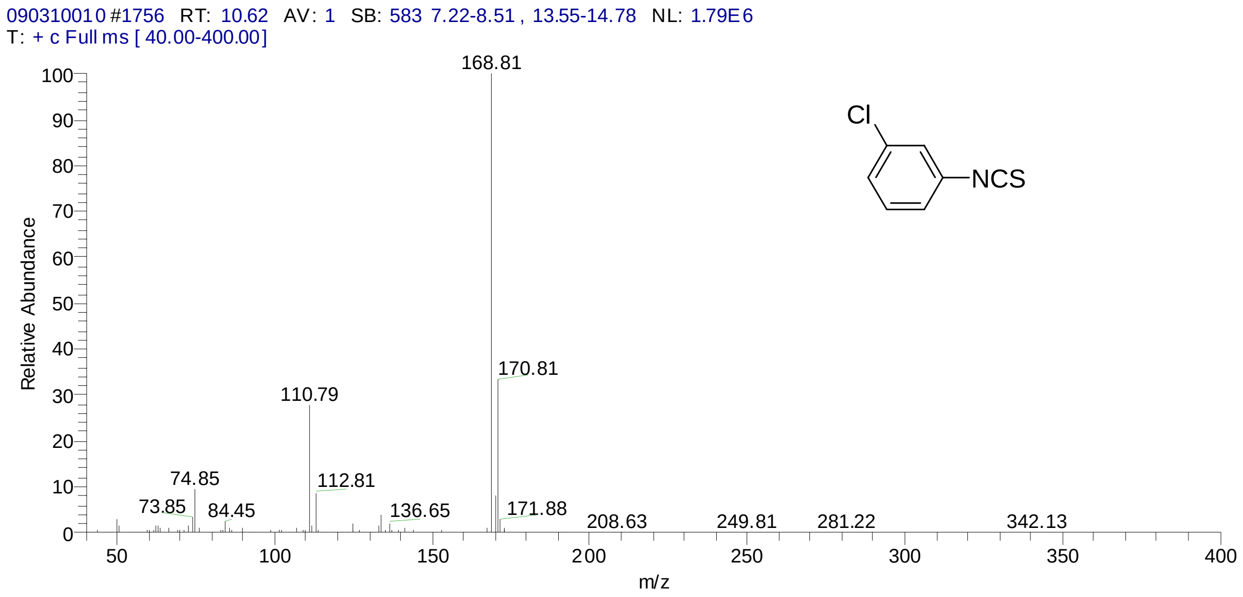
090310010 #1756 RT: 10.62 AV: 1 SB: 583 7.22-8.51 , 13.55-14.78 NL: 1.79E6
T: + c Full ms [40.00-400.00]
Relative Abundance
m/z
168.81
170.81
110.79
74.85
112.81
73.85
84.45
136.65
171.88
208.63
249.81
281.22
342.13
Cl
NCS

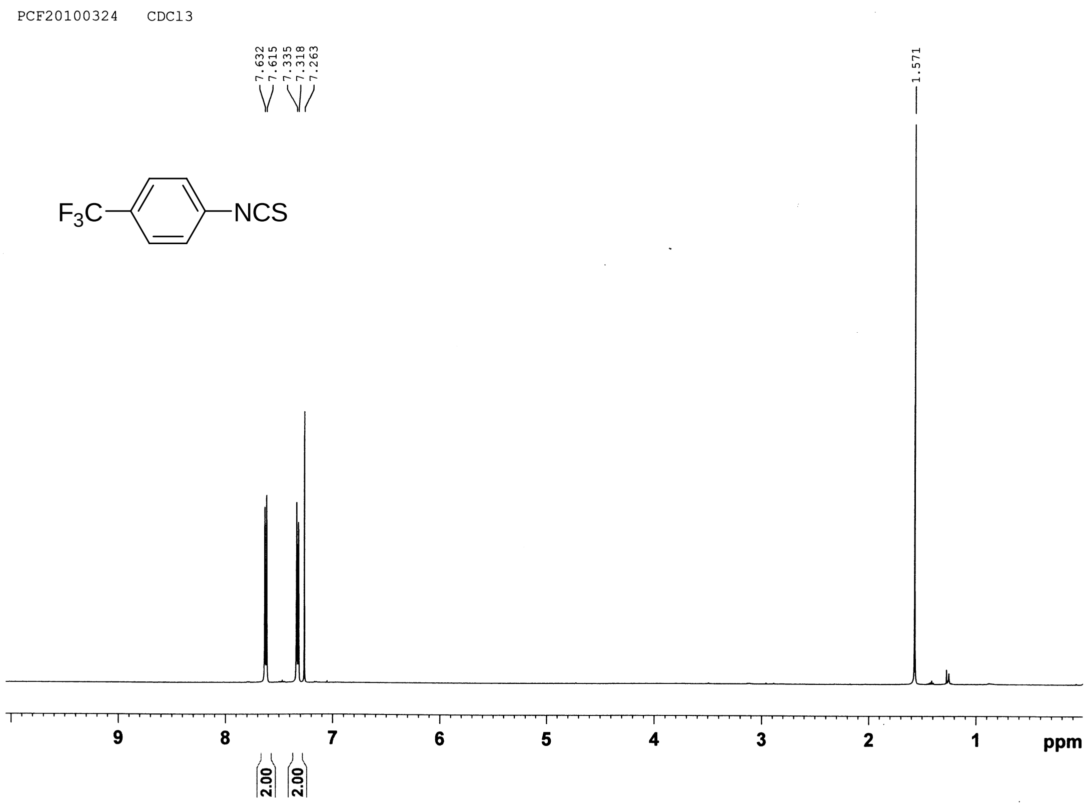

PCF20100324 CDCl3
7.632
7.615
7.335
7.318
7.263
1.571
F_3C
NCS
9
8
7
6
5
4
3
2
1
ppm
2.00
2.00

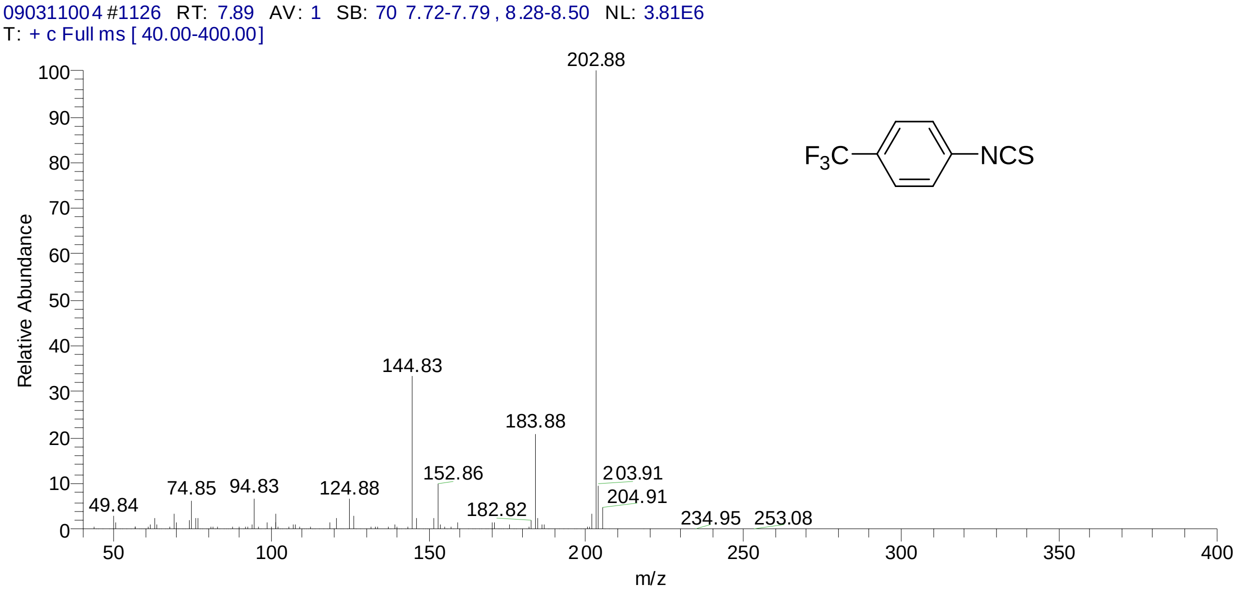

090311004 #1126 RT: 7.89 AV: 1 SB: 70 7.72-7.79 , 8.28-8.50 NL: 3.81E6
T: + c Full ms [40.00-400.00]
Relative Abundance
m/z
202.88
144.83
183.88
152.86
203.91
204.91
49.84
74.85
94.83
124.88
182.82
234.95
253.08
F_3C
NCS

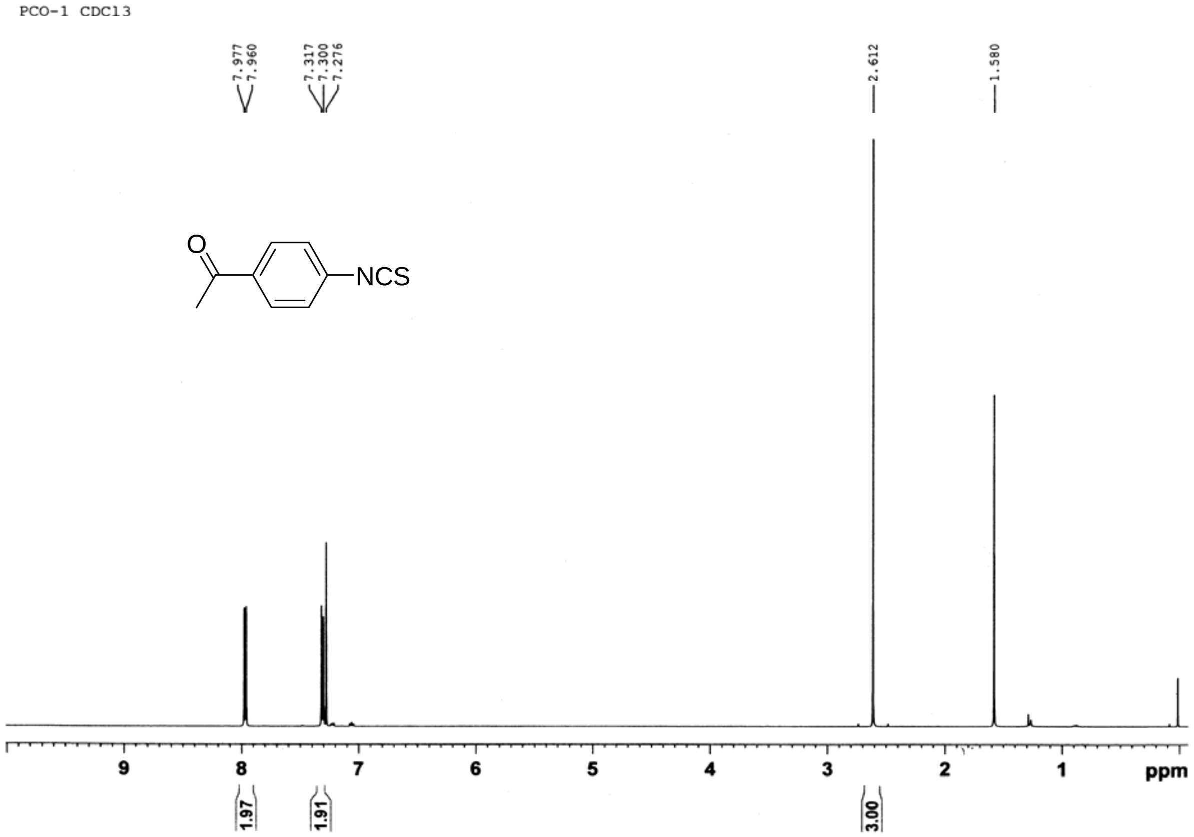
PCO-1 CDCl3
7.977
7.960
7.317
7.300
7.276
2.612
1.580
NCS
9
8
7
6
5
4
3
2
1
ppm
1.97
1.91
3.00

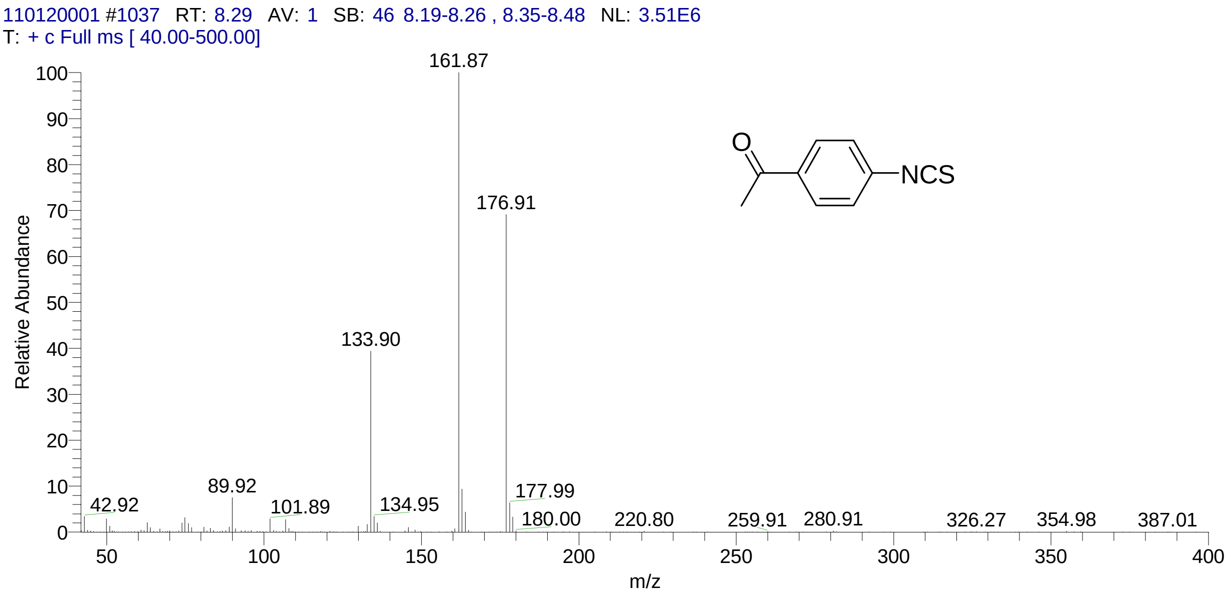
110120001 #1037 RT: 8.29 AV: 1 SB: 46 8.19-8.26 , 8.35-8.48 NL: 3.51E6
T: + c Full ms [40.00-500.00]
Relative Abundance
m/z
161.87
176.91
133.90
89.92
42.92
101.89
134.95
177.99
180.00
220.80
259.91
280.91
326.27
354.98
387.01
NCS

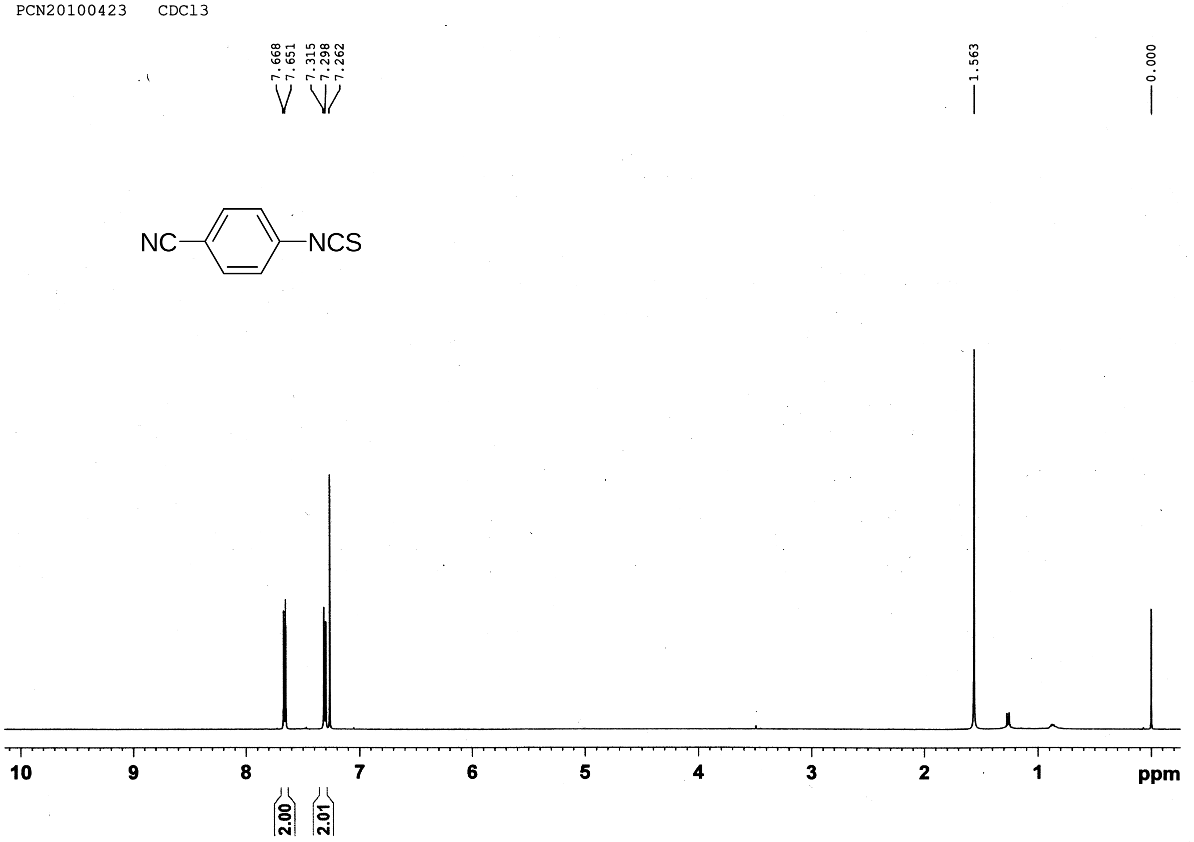

PCN20100423 CDCl3
7.668
7.651
7.315
7.298
7.262
1.563
0.000
NC
NCS
10
9
8
7
6
5
4
3
2
1
ppm
2.00
2.01

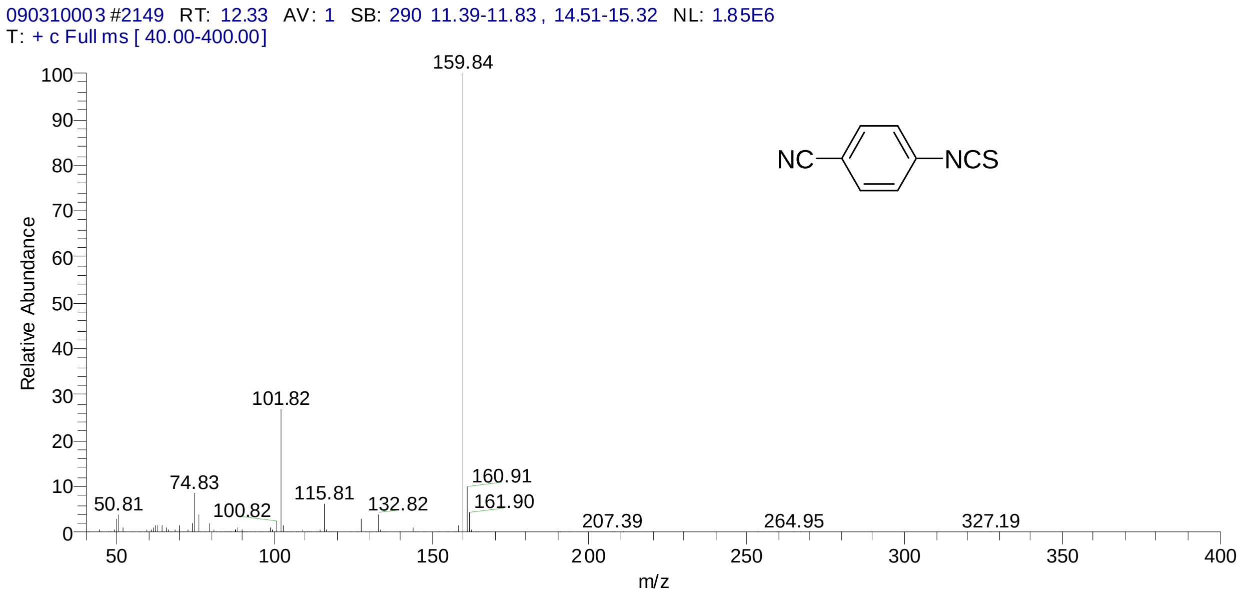

090310003 #2149 RT: 12.33 AV: 1 SB: 290 11.39-11.83 , 14.51-15.32 NL: 1.85E6
T: + c Full ms [40.00-400.00]
Relative Abundance
m/z
159.84
101.82
74.83
50.81
100.82
115.81
132.82
160.91
161.90
207.39
264.95
327.19
NC
NCS

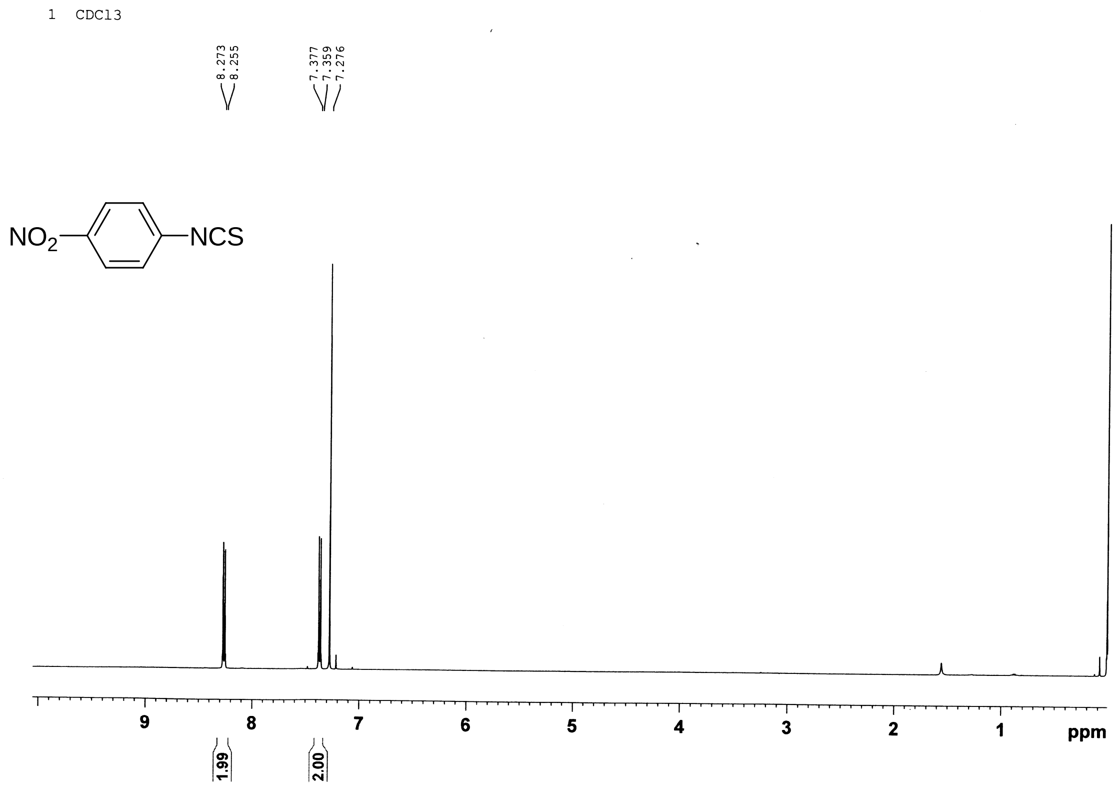
1 CDCl3
8.273
8.255
7.377
7.359
7.276
NO2
NCS
9
8
7
6
5
4
3
2
1
ppm
1.99
2.00

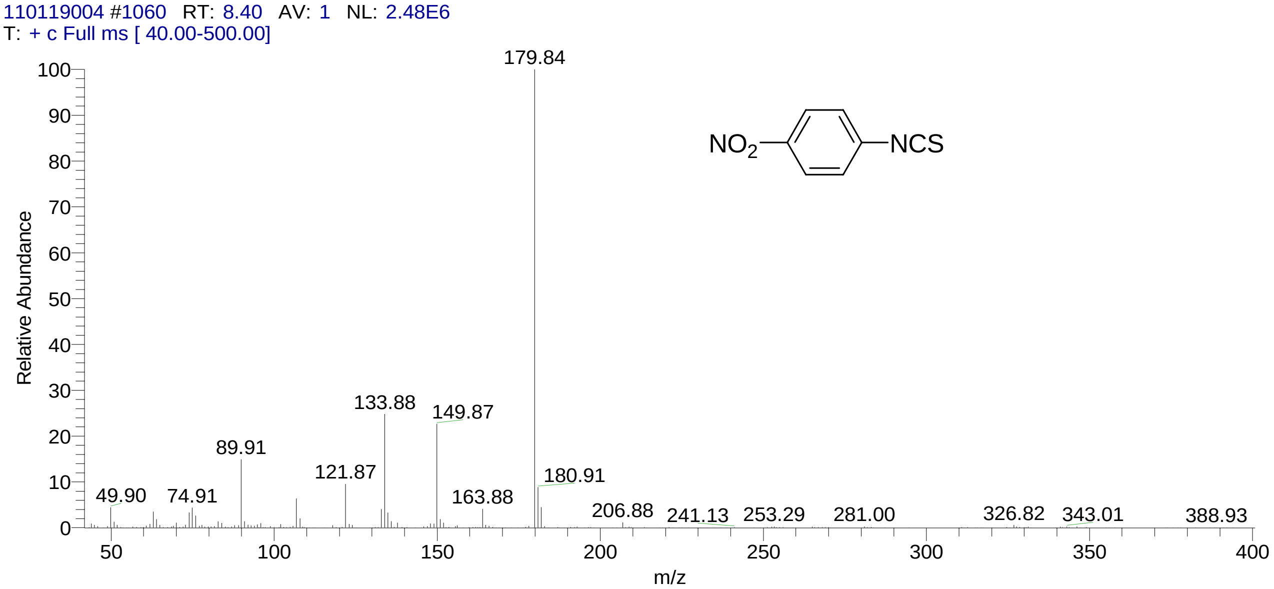
110119004 #1060 RT: 8.40 AV: 1 NL: 2.48E6
T: + c Full ms [40.00-500.00]
179.84
133.88
149.87
89.91
121.87
180.91
49.90
74.91
163.88
206.88
241.13
253.29
281.00
326.82
343.01
388.93
Relative Abundance
m/z
NO2
NCS

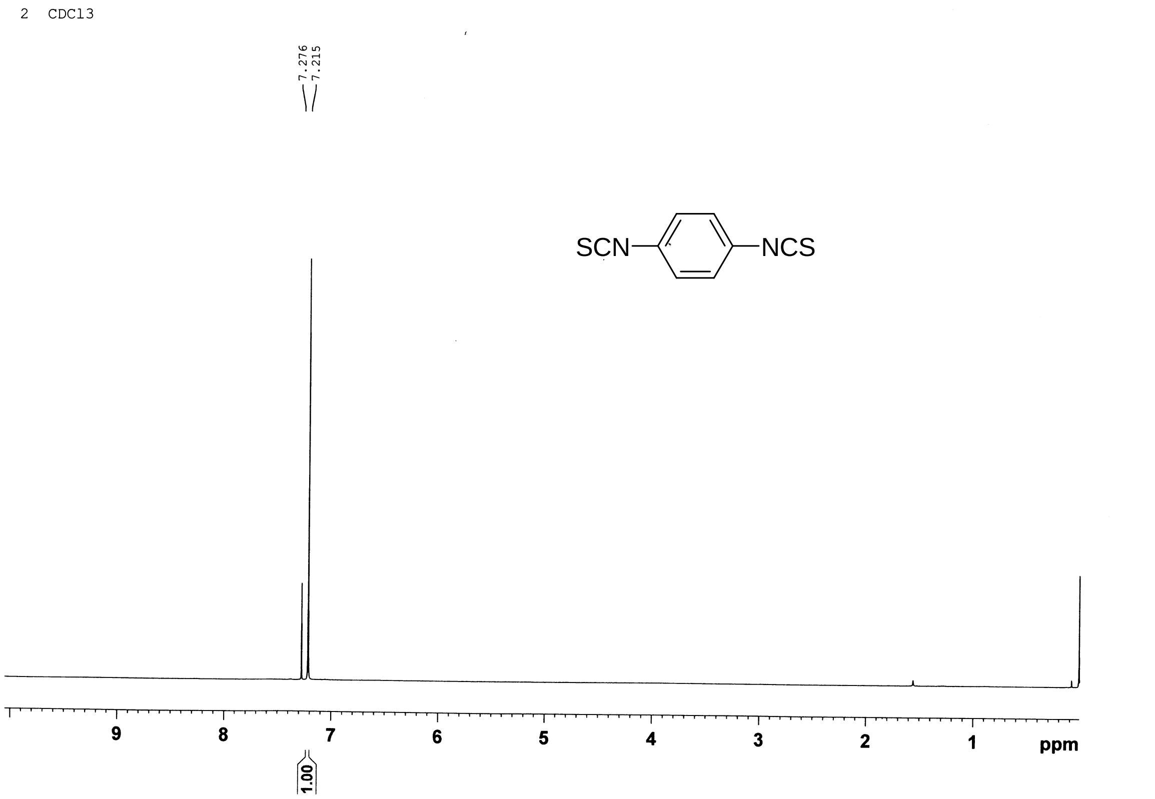
2 CDCl3
7.276
7.215
SCN
NCS
9
8
7
6
5
4
3
2
1
ppm
1.00

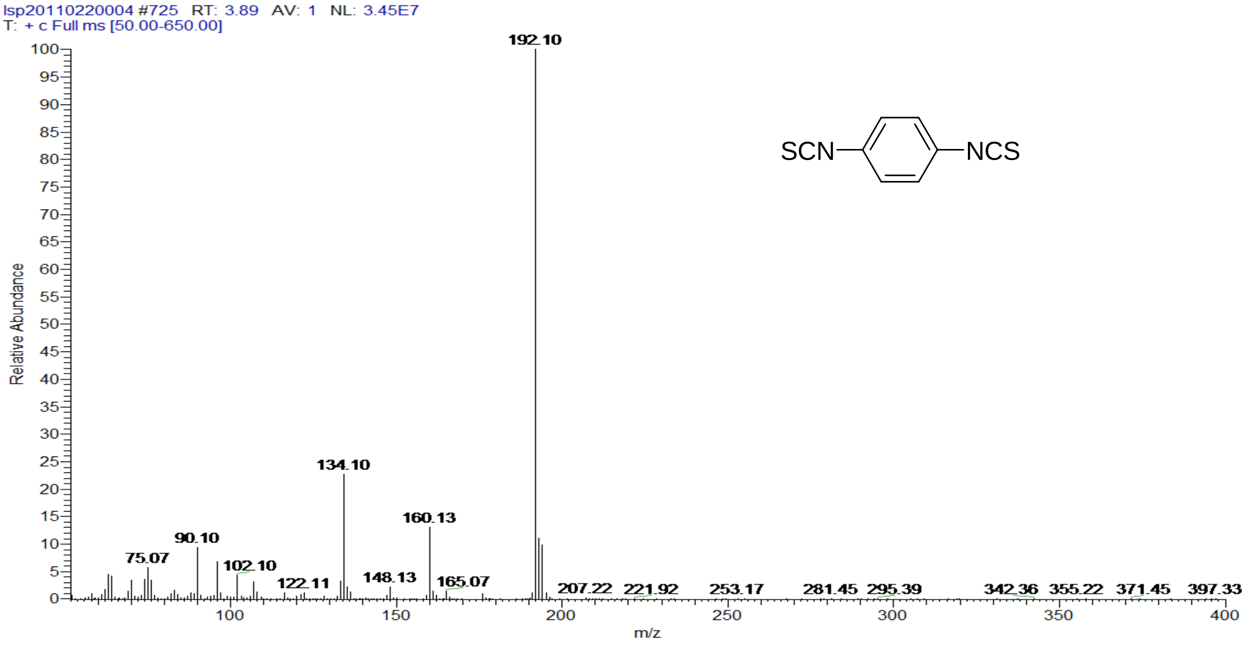
lsp20110220004 #725 RT: 3.89 AV: 1 NL: 3.45E7
T: + c Full ms [50.00-650.00]
192.10
SCN
NCS
Relative Abundance
134.10
160.13
90.10
75.07
102.10
122.11
148.13
165.07
207.22
221.92
253.17
281.45
295.39
342.36
355.22
371.45
397.33
m/z
